# Comparative single-cell transcriptional atlases of *Babesia* species reveal conserved and species-specific expression profiles

**DOI:** 10.1371/journal.pbio.3001816

**Published:** 2022-09-22

**Authors:** Yasaman Rezvani, Caroline D. Keroack, Brendan Elsworth, Argenis Arriojas, Marc-Jan Gubbels, Manoj T. Duraisingh, Kourosh Zarringhalam

**Affiliations:** 1 Department of Mathematics, University of Massachusetts Boston, Boston, Massachusetts, United States of America; 2 Department of Immunology and Infectious Diseases, Harvard T.H. Chan School of Public Health, Harvard University, Boston, Massachusetts, United States of America; 3 Department of Physics, University of Massachusetts Boston, Boston, Massachusetts, United States of America; 4 Department of Biology, Boston College, Chestnut Hill, Massachusetts, United States of America; 5 Center for Personalized Cancer Therapy, University of Massachusetts Boston, Boston, Massachusetts, United States of America; University of South Florida, UNITED STATES

## Abstract

*Babesia* is a genus of apicomplexan parasites that infect red blood cells in vertebrate hosts. Pathology occurs during rapid replication cycles in the asexual blood stage of infection. Current knowledge of *Babesia* replication cycle progression and regulation is limited and relies mostly on comparative studies with related parasites. Due to limitations in synchronizing *Babesia* parasites, fine-scale time-course transcriptomic resources are not readily available. Single-cell transcriptomics provides a powerful unbiased alternative for profiling asynchronous cell populations. Here, we applied single-cell RNA sequencing to 3 *Babesia* species (*B*. *divergens*, *B*. *bovis*, and *B*. *bigemina*). We used analytical approaches and algorithms to map the replication cycle and construct pseudo-synchronized time-course gene expression profiles. We identify clusters of co-expressed genes showing “just-in-time” expression profiles, with gradually cascading peaks throughout asexual development. Moreover, clustering analysis of reconstructed gene curves reveals coordinated timing of peak expression in epigenetic markers and transcription factors. Using a regularized Gaussian graphical model, we reconstructed co-expression networks and identified conserved and species-specific nodes. Motif analysis of a co-expression interactome of AP2 transcription factors identified specific motifs previously reported to play a role in DNA replication in *Plasmodium* species. Finally, we present an interactive web application to visualize and interactively explore the datasets.

## Abstract 2

Babesia is a genus of apicomplexan parasites that infect vertebrate red blood cells, but difficulties in synchronizing the parasites has limited knowledge of the Babesia replication cycle. This study uses single-cell RNA sequencing of three Babesia species to map the replication cycle and construct pseudo-synchronized time-course gene expression profiles.

## Introduction

Apicomplexan parasites of the genus *Babesia* are some of the most widespread blood parasites of vertebrates, second only to the trypanosomes [1]. Babesiosis has long been recognized as a disease of tremendous veterinary and agriculture importance, causing hundreds of millions of dollars of economic losses every year [2,3]. Since the first reported case of human babesiosis, caused by *B*. *divergens*, reported in 1956, and compounded by the emergence of *B*. *microti* in the US, babesiosis has steadily been gaining recognition as an important human parasitic disease [4–7]. The disease can range from mild febrile illness to severe, life-threatening disease, particularly in immunocompromised patients [6,8]. *Babesia* is usually transmitted through the bite of an infected tick [9], but can also be transmitted congenitally and via blood transfusion [10–12]. Indeed, *Babesia* is listed as a top priority pathogen in the blood supply [13]. Beyond human pathogens, there are at least 100 species of *Babesia* described that cause disease in a variety of hosts [6,14]. Bovine babesiosis, predominantly caused by *B*. *bovis*, *B*. *bigemina*, and *B*. *divergens*, is of significant concern, often resulting in fulminating infection and high mortality, leading to significant economic and agricultural losses [15,16]. With *Babesia* representing such a wide diversity of disease-causing parasites, identifying both conserved and divergent biology is essential to developing therapeutic and vaccine interventions.

In the asexual replicative cycle, *Babesia* species are obligate intracellular parasites that infect red blood cells (RBCs). While knowledge about the morphology of *Babesia* parasites during these division cycles exists, molecular details of the asexual replication cycle are limited. Transcriptomic studies have been done on various *Babesia* spp. populations to profile life stage, egress and invasion, and virulence [17–22]. While these studies provide rich data resources, no transcriptomic data have been generated to comprehensively describe the asexual replication cycle, as has been done in other related parasites [23–27]. Most knowledge of the molecular mediators of the *Babesia* spp. replication cycle has been gleaned through comparative approaches with *Plasmodium* spp. and *Toxoplasma gondii* [28], and through the analysis of genomic sequences [17,18,29–36]. Currently, synchronization of *B*. *divergens* and *B*. *bovis* relies on mechanical release of parasites from the RBC, with other species still unable to be synchronized [37,38]. The current methods of synchronization are variable and do not yet allow for tightly synchronized populations of parasites. Consequently, to the best of our knowledge, only a single synchronous transcriptomic dataset exists to date [39]. While single-cell RNA sequencing (scRNA-seq) can overcome the difficulties of synchronization, no such data have yet been generated for *Babesia* species.

Due to these gaps in knowledge, and the limitations of synchronization, scRNA-seq presents a promising method for delineating the *Babesia* intraerythrocytic replication cycle at a fine scale. This approach offers a powerful, unbiased method of profiling heterogenous and asynchronous cell populations. scRNA-seq has been used successfully in a range of other apicomplexan parasites and has provided key insights into parasite biology not previously detectable using bulk RNA sequencing (RNA-seq) methods. In *Plasmodium* species, scRNA-seq has been used to describe cell populations through the entire life cycle, from the intraerythrocytic developmental cycle (IDC) through the mosquito [40–47]. This has led to the development of comprehensive cell atlases for both *P*. *berghei* [41] and *P*. *falciparum* [48]. Additionally, scRNA-seq in *T*. *gondii* was used to reveal novel regulators of life cycle stage progression [49,50]. Combined with the ability to culture many diverse species of *Babesia* in vitro [51,52], comparative scRNA-seq offers a unique opportunity in *Babesia* to identify core, conserved regulators of progression through the replicative cycle.

Here, we performed scRNA-seq on 3 bovine *Babesia* species that can be readily cultured in vitro: *B*. *bovis*, *B*. *bigemina*, and *B*. *divergens*. Additionally, to investigate a possible role of the host RBC on the replication cycle, we performed scRNA-seq on *B*. *divergens* adapted to either human RBCs or bovine RBCs in in vitro culture. Using these 4 datasets, we reconstructed the transcriptome of the asexual replication cycle for *Babesia* spp. and mapped the transition points of the developmental phases. We show that despite the evolutionary divergence of these *Babesia* species, the underlying expression patterns of their replication cycles are highly similar. Using these data, we were also able to reconstruct gene co-expression networks and quantify the interactomes of gene families important in each phase of development. Analysis of the interactomes reveals a core conserved set of replication cycle regulators. Taken together, these data represent, to our knowledge, the first asexual replication cycle atlases for any *Babesia* species, as well as the first single-cell transcriptomic datasets. To facilitate usage, we provide an interactive web application for visualization and exploration of our datasets. The web app provides functionality to profile gene expression, perform comparative transcriptomics analysis, assess and visualize gene expression timing across the species, and explore co-expression networks. The web app is available at https://umbibio.math.umb.edu/babesiasc/.

## Results

### scRNA-seq analysis reveals the characteristic cyclical pattern of expression in replicating *Babesia* blood-stage parasites

**Fig 1:**
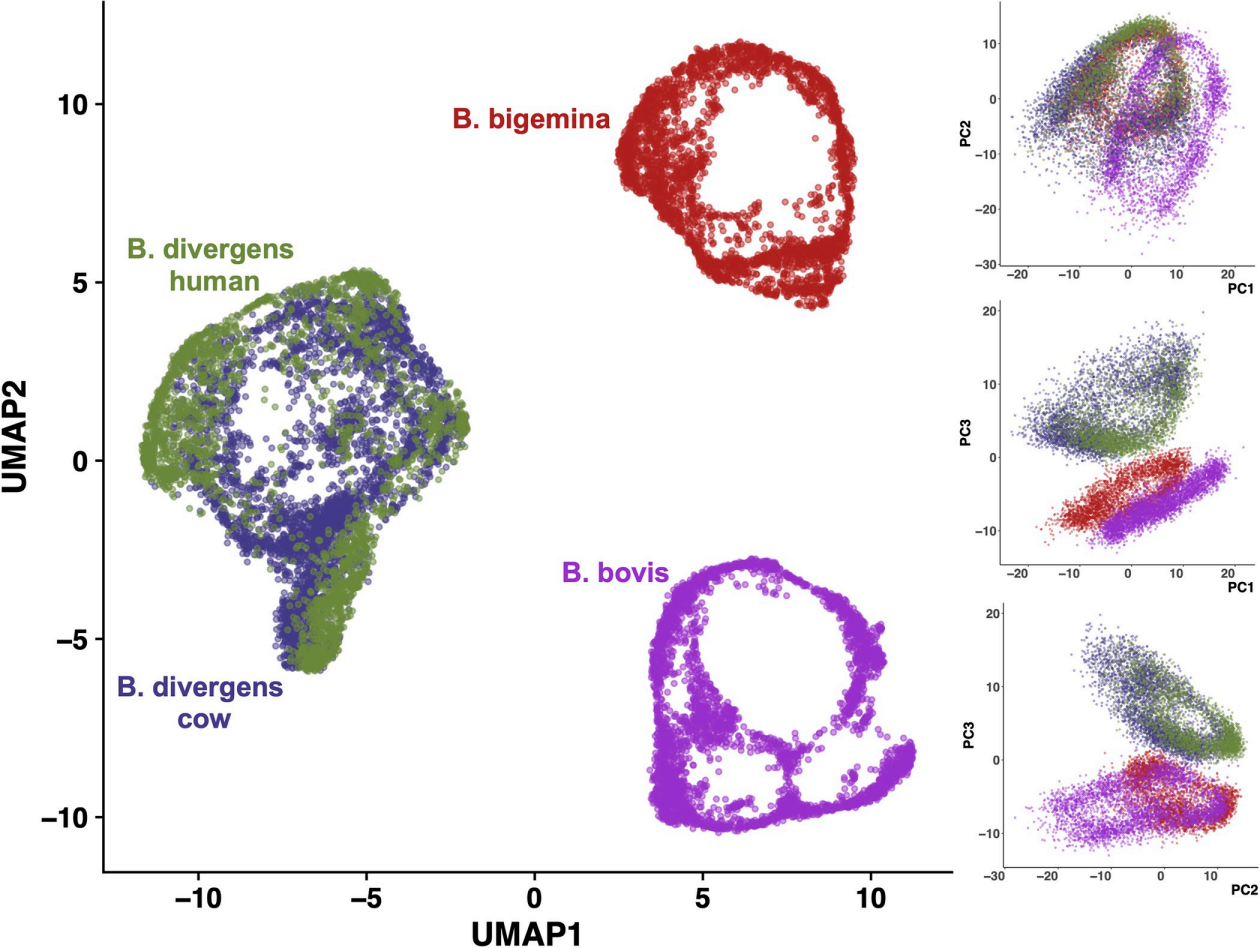
Projection of single-cell RNA sequencing data onto UMAP coordinates and the first 3 PC coordinates. *B*. *divergens* in human and bovine host cells cluster together. PCA, principal component; UMAP, Uniform Manifold Approximation and Projection. Data and code for generating the figure are available at https://github.com/umbibio/scBabesiaAtlases.

To characterize replication cycle progression in *Babesia* spp., we performed scRNA-seq on 3 *Babesia* species: *B*. *bigemina* and *B*. *bovis* of bovine origin, and *B*. *divergens* of human origin. To interrogate potential host-cell-specific differences, we collected *B*. *divergens* parasites growing in vitro in both bovine and human RBCs. The alignment rates were over 90% in all species. Downstream processing and alignment detected between 8,719 and 12,910 cells across the 4 species. The median number of genes detected per cell varied from 417 to 629. Additional filters were applied using the Seurat R package [53]. The number of cells and genes that passed the Seurat cutoffs varied from 3,200 to 4,000 and from 3,563 to 3,810, respectively, across the 3 species. All alignment and data quality metrics are available in S1 Table. Statistics on *Babesia* genomes regarding the percentage of exons, introns, and intergenic regions, as well as the proportions of overlapping genes, are provided in S2 Table. Expression datasets were combined using the expression of 2,548 orthologous genes obtained from reciprocal blast hits and visualized on Uniform Manifold Approximation and Projection (UMAP) and principal component analysis (PCA) coordinates (Fig 1). The projected data reveal a circular pattern shared across all *Babesia* species. that is characteristic of asexual replication in both *T*. *gondii* and *Plasmodium* spp. [23,41,50]. This circular pattern of expression is a consequence of periodicity in gene expression during the replication cycle and the existence of clusters of co-expressed genes. In the UMAP projection, *B*. *divergens* in human host cells and *B*. *divergens* in bovine host cells cluster together, indicating that the different hosts result in relatively few changes overall compared to the differences between *Babesia* species. The same analysis was repeated for each dataset independently using all genes, and also showed a circular pattern (Fig A in S1 Text).

### Pseudo-time analysis maps the progression of gene expression and transition points in the asexual replicative cycle

**Fig 2:**
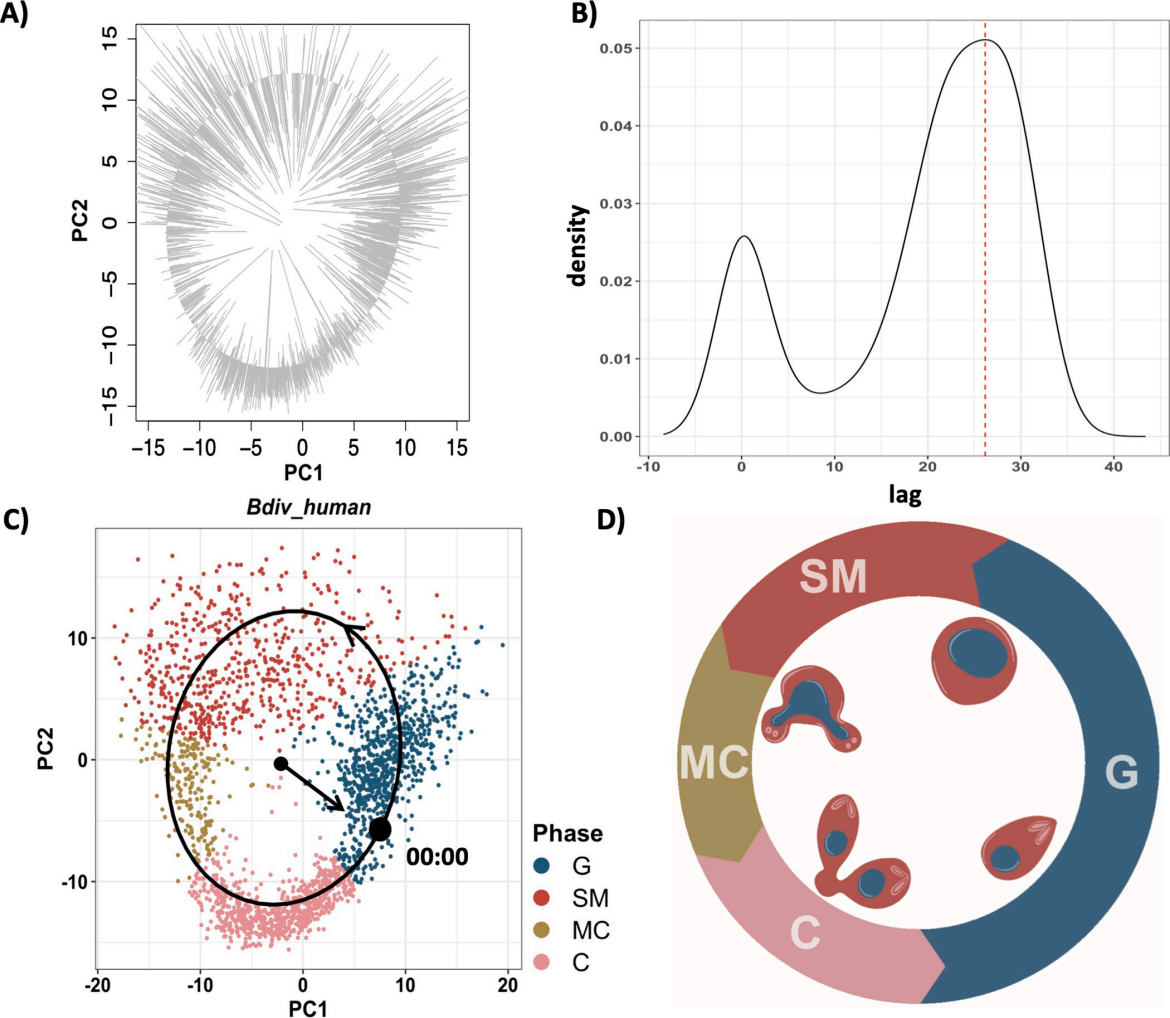
Pseudo-time reconstruction. (A) Cells were orthogonally projected on the elliptic principal curve fitted to the first 2 PCA coordinates and ordered sequentially using a random start point. The pseudo-time gene curve was constructed by mapping the cell orders to 0–12 h. (B) Distribution of the lag time between pseudo-time gene curves and matched gene curves from synchronized bulk measurements. Lag time was calculated using cross-correlation. The bimodal distribution indicates 2 optimal lag times corresponding to monotonic curves (lag 0) and cyclic curves (red dashed line). (C) PCA plot of *B*. *divergens* in human RBCs. Start time (black dot) was adjusted according to the optimal lag time corresponding to the larger peak in (B). The black arrow on the principal elliptic curve indicates the flow of time. Transition points were mapped to the PCA coordinates in *B*. *divergens* in human RBCs, and the colors represent each inferred phase. (D) Schematic representation of the *Babesia* spp. cell division cycle in RBCs. PC, principal component; PCA, principal component analysis; RBC, red blood cell. Data and code for generating the figure are available at https://github.com/umbibio/scBabesiaAtlases.

To map the progression of gene expression during the replication cycle, we performed a pseudo-time analysis by fitting an elliptic principal curve to the first 2 PCA coordinates of *B*. *divergens* [54]. Cells were orthogonally projected onto the curve and ordered to mimic pseudo-time (Fig 2A). The start point was arbitrarily picked and set to 0 and the end point was set to 12 h [37,38], representing the approximate time of a division cycle in *Babesia* spp. [38,39]. We partitioned the projected cells along the pseudo-time course into 20-min time intervals. Cells in each partition were considered “synchronized replicates.” Expression of genes along the pseudo-time course were then used to construct gene expression curves. Genes whose expression did not show temporal correlation with pseudo-time were identified by fitting a generalized additive model (GAM) and calculating the goodness of fit (adjusted *p*-value < 0.01). These include constitutively expressed genes as well as genes that are sporadically and randomly expressed. Depending on the dataset, a total of 2,504–2,741 genes passed the criteria and were retained for further analysis (S1 Table). It should be noted that the scale of pseudo-time differs from that of the actual cell division cycle time. Information on the actual span of cell cycle phases is needed to match the 2 through piecewise linear scaling. However, this scaling has no impact on the subsequent analysis of relative timing of expression, identification of differentially expressed genes (DEGs), or clustering of gene curves.

To adjust the start time and map the direction of replicative cycle progression, we utilized gene expression data from synchronized bulk RNA-seq from *B*. *divergens* [39]. The time course data consisted of 7 bulk RNA-seq measurements of synchronized *B*. *divergens* parasites over a 12-h period. A total number of 3,993 genes were detected in the synchronized data, of which 2,236 showed significant expression changes over the time period (adjusted *p*-value < 0.01). We calculated the cross-correlation of gene expression curves in single cells and the corresponding genes in synchronized bulk experiments and identified the lag time that maximized the correlation. Essentially, in the cross-correlation analysis, we shift the start point of the expression curve in the scRNA-seq data and calculate the correlation with the bulk data. This is done for each gene independently, and the lag time maximizing the cross-correlation is calculated. Fig 2B shows the lag time distribution across all genes in *B*. *divergens*. The 2 distinct peaks correspond to the optimal shift for monotonically expressed genes (no lag) and peaking genes (lag at 28). The lag corresponding to the second peak was used to adjust the lag time across all genes and to set the start point of the replication cycle clock in single cells (Fig 2C). Fig B in S1 Text shows a few example curves of genes corresponding to the optimal lag time, as well as genes corresponding to the lag time 0.

To identify the transition points through the replication cycle of *Babesia* spp., we used previously assigned replication cycle genes from *T*. *gondii*. Although transition points can be inferred computationally, we reasoned that *T*. *gondii* would be most informative because of its similar binary division modes shared with *Babesia* spp. and because the *T*. *gondii* phases of the cell division cycle were identified using DNA content analysis [50]. Moreover, computationally inferred phases generally agree with *T*. *gondii* inferred phases (Fig C in S1 Text). There is a high correlation between peak expression time of DEGs and the replicative cycle in *T*. *gondii* (Fig D in S1 Text). The timing of peak expression of the *Babesia* spp. orthologous genes of the top 20 *T*. *gondii* replicative cycle marker genes was used to map transition time points. There is an overlap between the distribution of peak time expression of S and M as well as M and C markers (Fig D in S1 Text). Further information on mapping the transition points can be found in S1 Text.

Based on this, we opted to define 4 “inferred” phases labeled as G, SM, MC, and C and marked the transitions to maximize the separation between the phases. These transition time points were used in pseudo-time to infer developmental phase transition points in *Babesia* spp. Fig 2C shows the PCA projection of *B*. *divergens* in human host cells, with the colors indicating the “inferred” phases, accompanied by a schematic depiction of the replication cycle (Fig 2D). These results show that by leveraging the geometry of gene expression data in asynchronously dividing parasites, single-cell gene expression can be converted to “pseudo-synchronized” time-course data at a fine resolution. This approach can be generally used to overcome some of the limitations of time-consuming and costly time-course experiments.

### Gene expression is highly correlated between synchronized bulk and pseudo-synchronized single-cell sequencing

**Fig 3:**
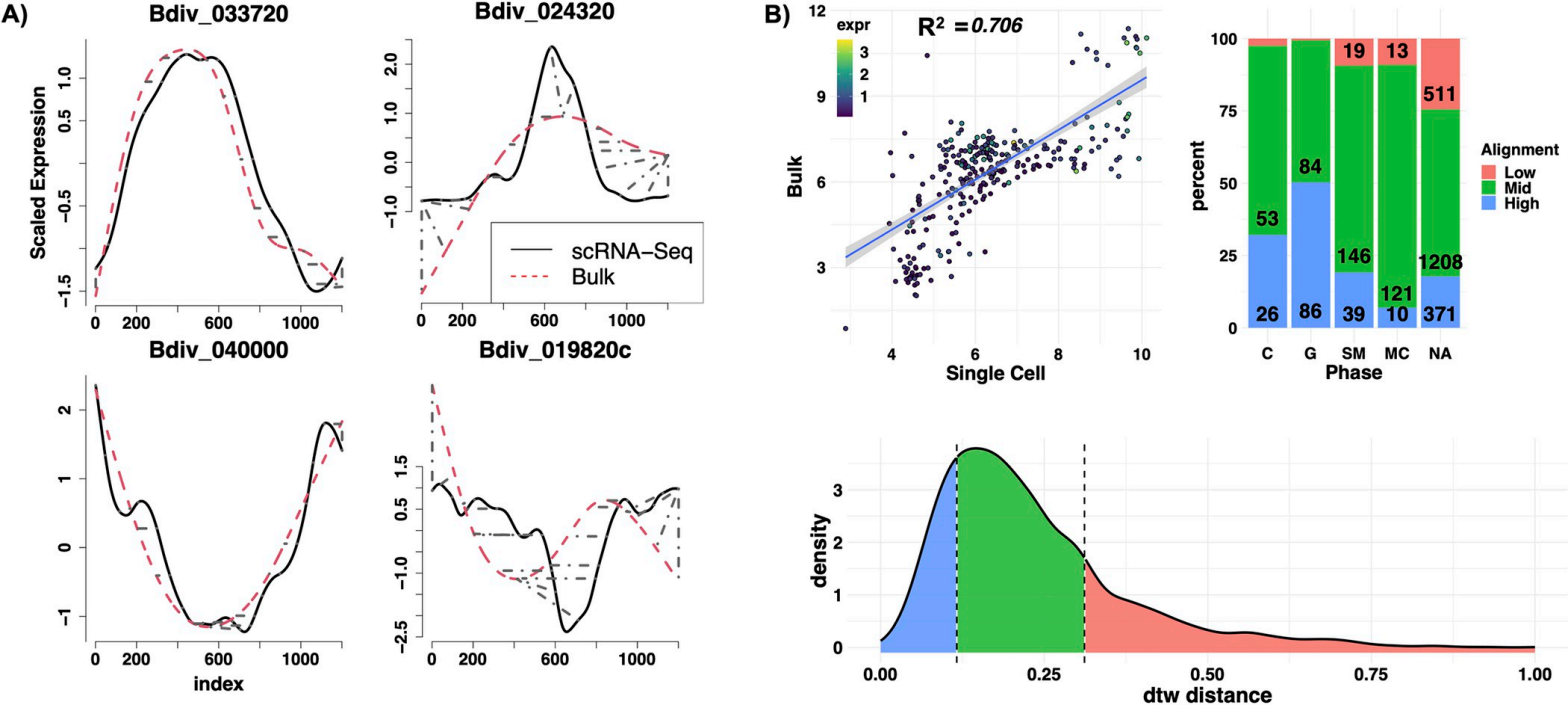
Quantification of alignment of gene curves between synchronized bulk and single-cell sequencing. (A) Dynamic time warping alignment of a sample gene: high alignments (left), low alignments (right). Gray dashed lines indicate the matched time index (warping). (B) Top left: Correlation between calculated peak times in S/M/C phases, excluding the boundary. Top right: Percent alignment distance categorized into high (distance < lower 20th percentile), low (distance > upper 80th percentile), and mid. Curves with no peak contain a higher percentage of poor alignment. Bottom: Distribution of dynamic time warping (dtw) alignment distance between single-cell and bulk sequencing. Shaded areas show boundaries of alignment categories. NA, not available. Data and code for generating the figure are available at https://github.com/umbibio/scBabesiaAtlases.

Using the newly inferred phases, we sought to assess the correlation of peak time as well as overall similarity of gene curves in the single-cell and synchronized bulk data. First, we fitted the curves with smoothing splines and interpolated points at regular intervals, aligned the scRNA-seq and bulk curves [55], and measured the distance between the curves (Materials and Methods). Time warping was applied to account for scaling differences between pseudo-time and real cell division time. Fig 3A shows some representative examples of high (left) and low (right) alignment for genes that peak (top) or deplete (bottom) in inferred SM phase. Next, we calculated peak expression time for each gene in both datasets. The peak assignment was limited to markers of S/M/C phases, where genes tend to have a more defined peak that lasts for a short period of time. The boundary cases of transitioning from C back to G were excluded, as marker genes assigned to G tend to have a flat expression period, followed by depletion at SM phase that slightly upticks in late C and transitions back to G. Fig 3B (top left) shows the correlation between peak expression times for S/M/C genes. The overall normalized alignment distance as measured by the dynamic time warping (DTW) algorithm was categorized into high, mid, and low, based on the distribution of the alignment distance (Fig 3B, top right and bottom). Overall, the data show that there is a high level of agreement between reconstructed pseudo-time in single-cell and synchronized time-course data, both in peak time expression as well as overall alignment. The bulk RNA-seq was used as a calibration point to set the start of the cell cycle in scRNA-seq and to validate the reconstructed expression curve, and played no role in subsequent analysis.

### Differential gene expression analysis of inferred phases

**Fig 4:**
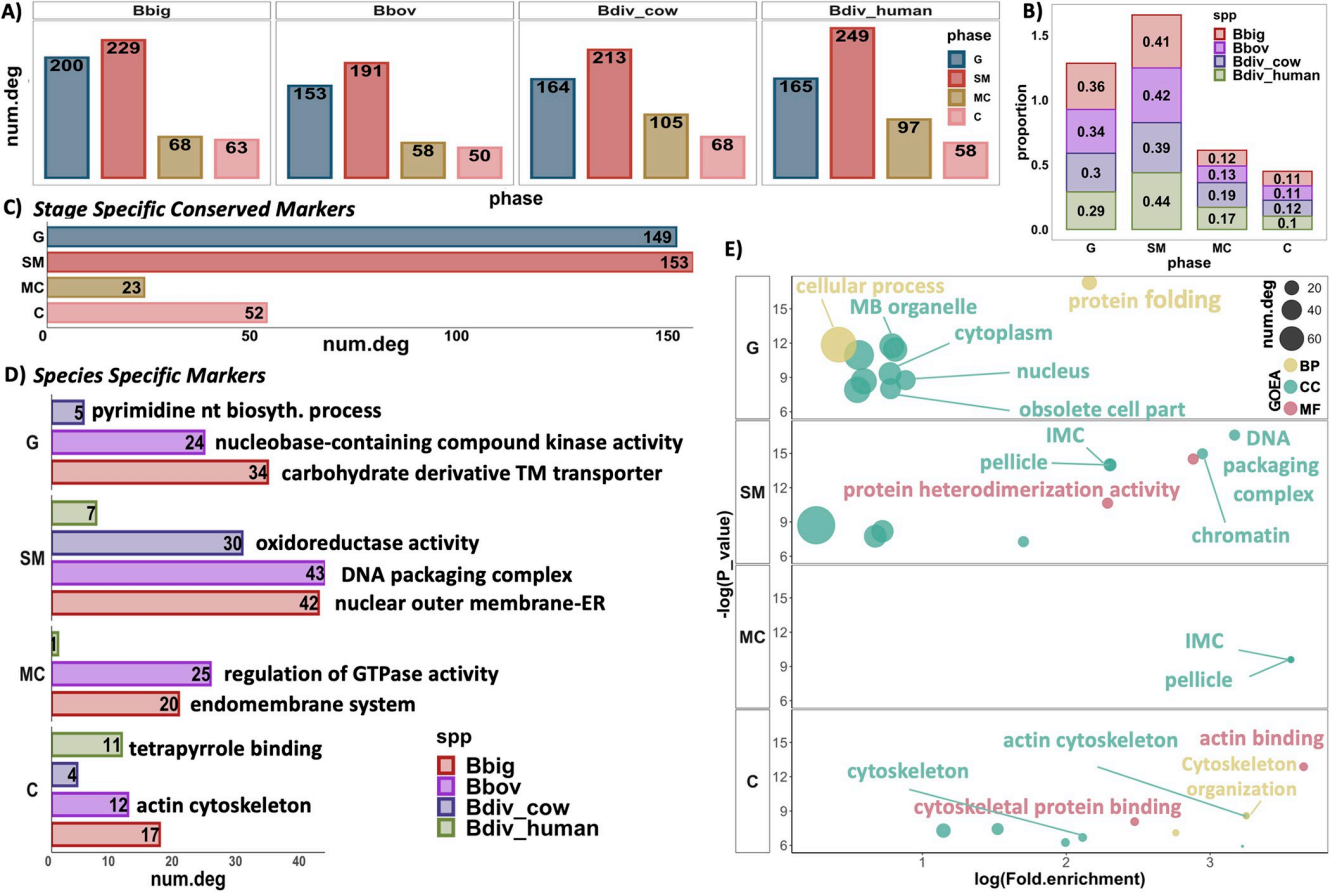
Differential expression and Gene Ontology enrichment analysis (GOEA). (A) Total number of inferred replication cycle differentially expressed genes (DEGs) in each species independent of other species. (B) Stack bar plot showing proportion of DEGs in each phase in all species. (C) Bar plot showing the total number of conserved DEGs of inferred replication cycle phases (rows) across all species. (D) Total number of DEGs of the indicated phase unique to the indicated species (colors). The most significant Gene Ontology (GO) term (Benjamini < 0.1) associated with the set of DEGs (each bar) is presented next to the bar. (E) Significant GO terms associated with conserved DEGs of the indicated phase (rows). Colors indicate the GO term category. Fold change > 2 and adjusted *p*-value < 0.01 were applied as cutoffs for all DEG analyses performed in this section. Note: All significant DEGs and GO hits can be found in S3 and S4 Tables. Data and code for generating the figure are available at https://github.com/umbibio/scBabesiaAtlases.

We performed differential gene expression analysis to identify (1) cross-species conserved replication cycle DEGs, (2) species-specific phase DEGs (i.e., replication cycle phase DEGs highly upregulated in 1 species), and (3) replication cycle phase DEGs of each species independent of other species (fold change [FC] > 2 and adjusted *p*-value < 0.01). The bar plots in Fig 4A, 4B, 4C, and 4D respectively represent the number of species-specific DEGs, the percentage of DEGs in each phase in each species, conserved replication cycle DEGs, and species-specific DEGs. Conserved DEGs were calculated using the built-in functions in the Seurat R package (Materials and Methods), which is more lenient than calculating conserved DEGs (and species-specific DEGs) by taking the intersection (and set difference) of DEGs across species. We repeated this analysis using intersection and set differences as well (Fig E in S1 Text). There is a minor difference between total number of DEGs detected; however, the overall results regarding the enrichment analysis are similar. Full lists of DEGs are provided in S3 Table. We performed a Gene Ontology enrichment analysis (GOEA) to investigate biological functions linked to each set of DEGs. For this analysis, genes were mapped to their *T*. *gondii* orthologs, and enrichment analysis was performed using the available Gene Ontology (GO) terms on ToxoDB. GO term and gene set categories with fewer than 10 genes were excluded from this analysis. Multiple principal significant GO terms are highlighted for the conserved genes in Fig 4E. All significant GO term hits can be found in S4 Table. The top-ranked GO term for the C inferred phase across the *Babesia* species is actin cytoskeleton, including several genes involved in actin polymerization (profilin, Bdiv_003910c; actin depolymerizing factor [ADF], Bdiv_021160), as well as myosin A (Bdiv_001770c). These genes are consistent with the cytoskeletal remodeling that occurs during cytokinesis. The SM and MC most highly enriched term relates to the pellicle. In *Babesia* parasites the pellicle is the structural site that organizes budding of daughter cells; thus, gene expression would need to occur prior to this process [56]. In *T*. *gondii*, expression of many of the orthologous genes peaks during the transition from S to M phase [23], which is consistent with the expression we observe here in *Babesia* spp. A major distinction between SM and MC inferred phase is the distinct upregulation of genes involved in biogenesis and segregation of the Golgi apparatus during SM phase—in mammalian cells this process is known to occur just prior to mitosis [57]. This is also in line with the previously observed timing of Golgi formation in *T*. *gondii*, where the Golgi undergoes elongation and segregation prior to the formation of the scaffolding of daughter cells (mitosis) [58]. Additionally, genes involved in vesicular transport and vesical formation (clathrin heavy chain, Bdiv_019640c; adaptin N terminal region family protein, Bdiv_009600c) were identified in SM phase. Finally, the genes identified as enriched in G phase are generally involved in organelle biogenesis including the apicoplast. In *P*. *falciparum*, apicoplast growth begins in the trophozoite phase prior to DNA replication [59]. Further, genes involved in protein folding, including several chaperones and heat shock proteins, are expressed in this stage. Genes involved in DNA replication start expression in late G phase (Fig F in S1 Text), suggesting that *Babesia* species follow a “just-in-time” expression pattern, in this case expressing genes needed during S phase DNA replication during G phase. Further details of phase-based DEGs can be found in S1 Text. These results show that the identified phases and the DEGs concur with known biology, although there is some degree of overlap between the phases, indicating the need for defining alternative transition points that more accurately reflect the progression of the cell cycle.

### Species-specific differential gene expression analysis

In addition to conserved DEGs of replication cycle progression, we also investigated species-specific DEGs of each inferred state using GO term enrichment, focusing on the most highly enriched processes (Fig 4D). The topmost significant GO term for each set of DEGs is shown next to the bar plot in Fig 4D. For each species, a different common thread emerges throughout their respective replicative cycle. Many of the enriched processes involve the endomembrane system and membrane transport in *B*. *bigemina*. These include nucleotide (ATP) transport (BBBOND_0211990), known to be important in mitochondrial transport in related parasites [60,61], and several genes with predicted function in the endomembrane system (BBBOND_0309310, BBBOND_0401740, BBBOND_0307820, BBBOND_0102060, BBBOND_0210875, BBBOND_0210880). However, in *B*. *bovis*, an emphasis on various signaling pathways emerges, including kinase activity (nucleoside diphosphate kinase family protein, BBOV_III005290; adenylate kinase, BBOV_IV002930) and hydrolase activity (putative GTPase activating protein for Arf, BBOV_IV012060; GTPase activator protein, BBOV_IV007530). For the latter, both enriched genes work to activate GTPases, which are known to be important regulators in other systems [62,63]. Species-specific enrichment in *B*. *divergens* mainly occurs in metabolic pathways, including fatty acid metabolism/pyrimidine biosynthesis (bovine: cytidine diphosphate-diacylglycerol synthase, Bdiv_002810c; human: choline/ethanolamine kinase, Bdiv_020970) and oxidoreductase activity (bovine: Bdiv_019910, Bdiv_030660, Bdiv_040430c). Taken together, these results suggest varying levels of dependence on vesicular transport, signaling, and metabolic pathways among *Babesia* species. Further description of differences by phase can be found in S1 Text. We also repeated the same analysis using a cutoff threshold of 1.5 for FC to test the robustness of the analysis (Fig G in S1 Text). As expected, the total number of identified DEGs increased, but the enrichment analysis was generally the same.

### Progression of gene expression during the replication cycle

**Fig 5:**
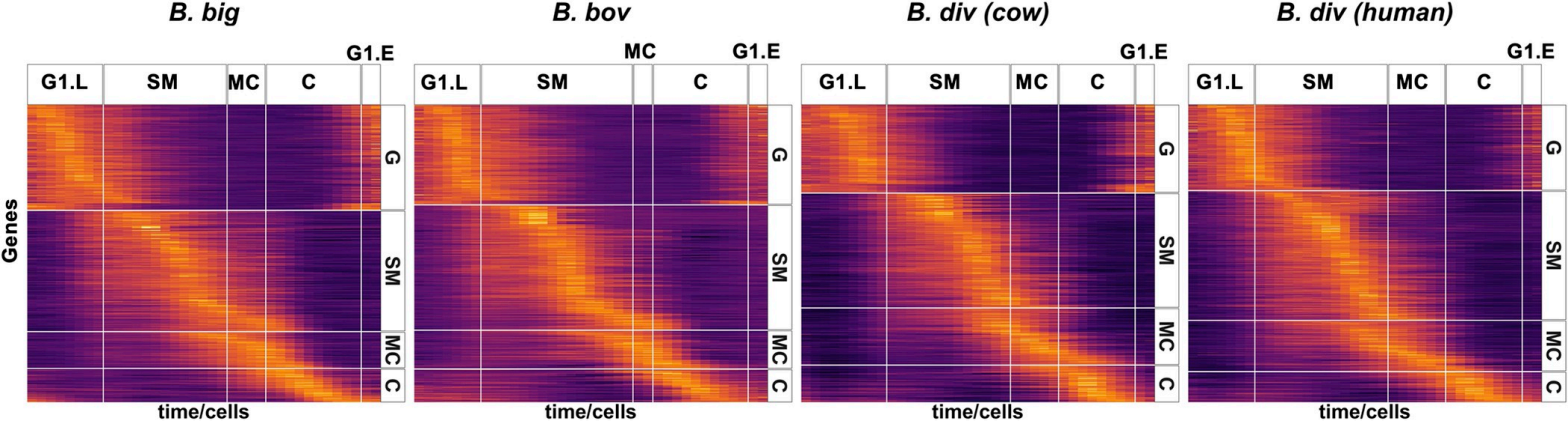
Transcription profile of replication cycle regulated genes: Heatmap of mean expression curves of differentially expressed genes of inferred replication cycle phases. Rows represent genes and columns represent pseudo-time-ordered cells. Horizontal facets group genes in the indicated phase. Vertical facets mark the transition time points. Analysis is done in each species independently (Fig 4A). Rows are ordered according to peak expression time. Data and code for generating the figure are available at https://github.com/umbibio/scBabesiaAtlases.

We sought to cluster and visualize the expression of DEGs during the replication cycle. DEGs of each phase were identified in each species independently (Fig 4A). We fitted smoothing B-splines to all gene curves and calculated the trends along the pseudo-time course. Fig 5 shows the heatmap of scaled expression of mean curves of the inferred replication cycle DEGs in each species. Genes are ordered by their peak expression time in each phase. Vertical lines show the mapped transition time points, while horizontal lines delineate the DEGs of each phase. The transition from C back to G consists of genes with “flat” peaks that cross the boundary. To better visualize this, we divided the G phase into late (G1 L) and early (G1 E), with G1 E directly proceeding the C phase. The heatmaps show that clusters of genes peak at similar times, with peak time expression gradually shifting through the replication cycle. Interestingly, there are visible points of transition in the peak expression time that seem to match well with cell division cycle phases, indicating that shifts in peak expression govern the progression of the cell division cycle. This “just-in-time” expression pattern profile is also seen in other parasite species [23,24,64–66], supporting the notion that replication cycle genes are organized into clusters of co-expressed/co-regulated genes that may be functionally related. A heatmap of 377 conserved replication cycle DEGs across all species (Fig 4C) ordered by their progression in *B*. *divergens* adapted to either human or bovine RBCs shows a more similar progression pattern compared to *B*. *bovis* or *B*. *bigemina* (Fig H in S1 Text), as also seen with the UMAP projection (Fig 1).

### Comparative differential expression analysis of *B*. *divergens* in human versus bovine RBCs

**Fig 6:**
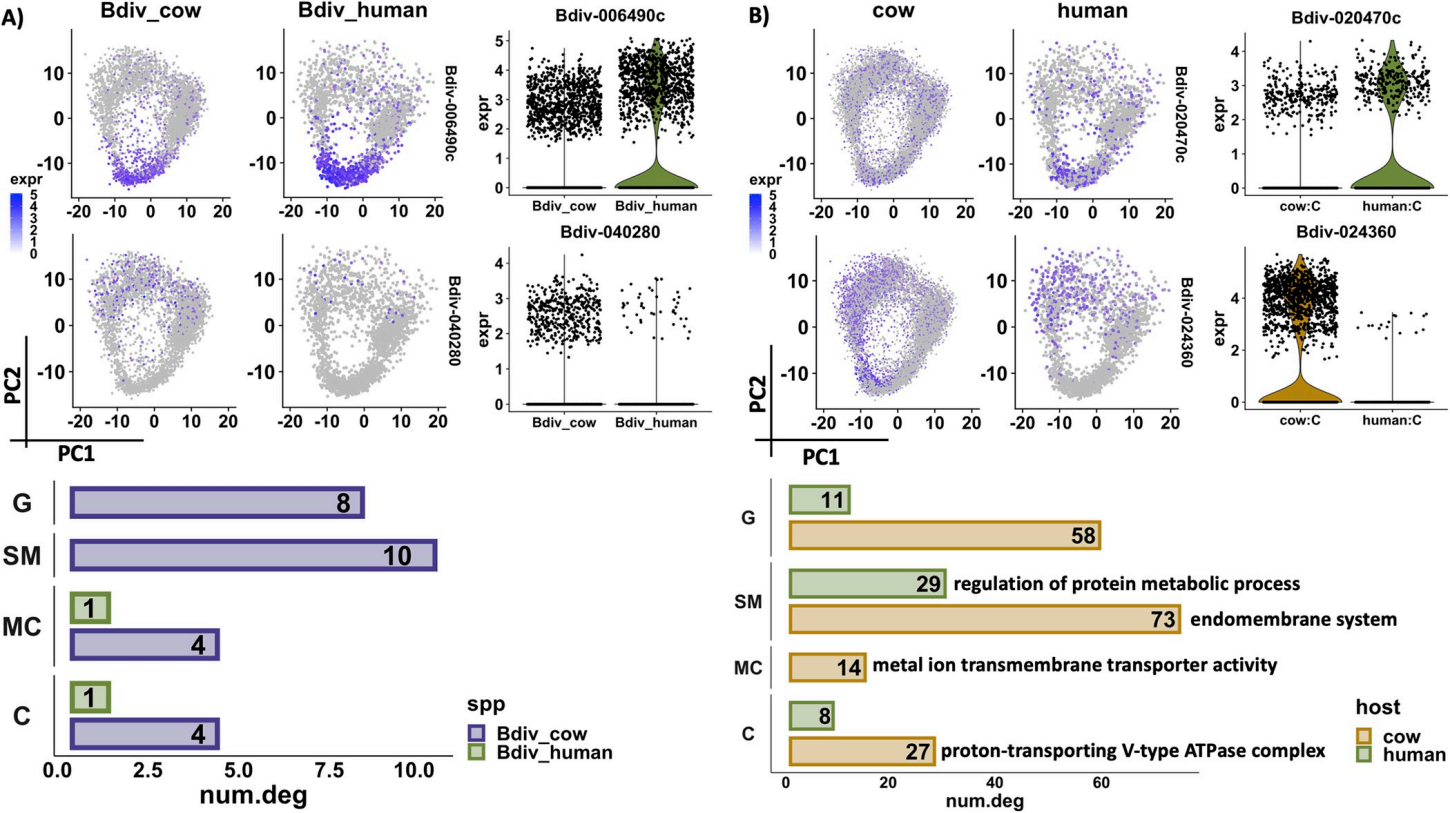
Host-specific differential expression and GO term enrichment analysis. (A) Top: Expression of top differentially expressed genes in *B*. *divergens* in bovine and human RBCs. Violin plots illustrate the distribution of expression of the top DEGs. Bottom: Bar plots showing the total number of DEGs in the indicated phase (rows) in each sample (colors). (B) Top: Expression of top DEGs in combined *B*. *bigemina*, *B*. *bovis*, and *B*. *divergens* in bovine RBCs versus *B*. *divergens* in human RBCs. Violin plots illustrate the distribution of expression of top DEGs. Bottom: Bar plots showing the total number of DEGs in the indicated phase (rows) in each sample (colors). To eliminate the confounding effect caused by species differences, any DEG between *B. divergens* and *B*. *bigemina* or between *B*. *divergens* and *B*. *bovis* in bovine RBCs was excluded from this analysis. The topmost significant GO term associated with each set of DEGs (Benjamini < 0.1) is presented next to the bar plot. DEG, differentially expressed gene; GO, Gene Ontology; PC, principal component; RBC, red blood cell. Data and code for generating the figure are available at https://github.com/umbibio/scBabesiaAtlases.

To investigate the impact of the host cell on the transcriptome, we performed a differential expression analysis followed by GO term enrichment and identified DEGs that are uniquely associated with growth in different host cells. First, we compared the transcriptome of *B*. *divergens* in human versus bovine RBCs in each of the inferred phases. This analysis quantifies species-specific host cell impact on the transcriptome. Fig 6A (top) shows the expression of the top DEG in human (Bdiv_006490c) and bovine (Bdiv_040280) RBCs. Bdiv_006490c is annotated as an aspartyl protease (*asp3*) that shares homology with plasmepsin X in *P*. *falciparum*, which is essential for egress and invasion of the parasites [67,68]. Bdiv_040280 is an unannotated protein that contains a mago binding domain. Mago domain containing proteins have been shown to be involved in post-transcriptional regulation in other parasites [69]. Mago proteins have also been shown to be involved in splicing and trafficking of mRNA [70]. The bar plot in Fig 6A (bottom) shows the total number of upregulated genes (FC > 1.5 and adjusted *p*-value < 0.01) specific to *B*. *divergens* in human RBCs and *B*. *divergens* in bovine RBCs in each phase. In total, we identified 28 genes across the inferred replication cycle phases that were differentially expressed between *B*. *divergens* grown in different host RBCs (S3 Table). The majority of changes observed occurred in metabolic pathways including lipid metabolism and pyrimidine biosynthesis (S1 Text). These underscore differences in nutrient transport and metabolism based on resident host cell.

Second, to quantify the impact of the host cell in a species-independent manner, we merged the transcriptomes for all *Babesia* species isolated from bovine RBCs and compared the merged transcriptome with that of *B*. *divergens* in human RBCs for each of the replication cycle phases. For this analysis, to ensure that parasite-specific differences did not confound the results, we excluded any gene that was differentially expressed between *B*. *divergens* in bovine RBCs and *B*. *bigemina* and *B*. *bovis* in bovine RBCs. Fig 6B (top) shows the expression of the top DEGs in human (Bdiv_020470c) and in bovine (Bdiv_024360) RBCs, projected on the PCA plot or shown using a violin plot to facilitate visualization of the expression distribution. The human DEG Bdiv_020470c is a cytochrome b5-like Heme/Steroid binding domain containing protein, while the bovine DEG Bdiv_024360 is a conserved hypothetical protein that contains a SNARE-associated Golgi protein domain as well as several transmembrane domains, which may be reflective of differences in membrane composition and nutrient availability between the host cells. The bar plot in Fig 6B (bottom) shows the total number of upregulated genes in parasites cultured in each host per phase as well as the top significant GO terms. GO terms associated with genes enriched in human-adapted *B*. *divergens* are mainly involved in protein kinase signaling (Bdiv_035640) and protein metabolism (Bdiv_022680, Bdiv_002540) during the C and SM phases. These findings suggest differences in protein dynamics/degradation and in signaling pathways in the parasites that reside in human RBCs.

In contrast to the human-adapted parasite, in the bovine-adapted parasites (*B*. *bigemina*, *B*. *bovis*, *B*. *divergens*) the majority of enriched GO terms are related to the Golgi apparatus, endomembrane system, and vesicular formation/transport (i.e., Bdiv_000390c, Bdiv_005170, Bdiv_009600c, Bdiv_018340, Bdiv_022710, Bdiv_028900c, Bdiv_032480), suggesting that vesicular transport and protein trafficking may be upregulated in parasites cultured in bovine RBCs. Further, there is an enrichment of genes involved in transport (i.e., Bdiv_010590, Bdiv_033310c). Taken together these results suggest that there are some differences in cellular processes of parasites propagated in bovine versus human RBCs that are driven by the host cell environment. Particularly, there appear to be consistent differences in protein metabolism, signaling, nutrient transport, and vesicular transport. A full list of DEGs and GO terms are available in S3 and S4 Tables and is described in S1 Text.

### Reconstructing the co-expression network

**Fig 7:**
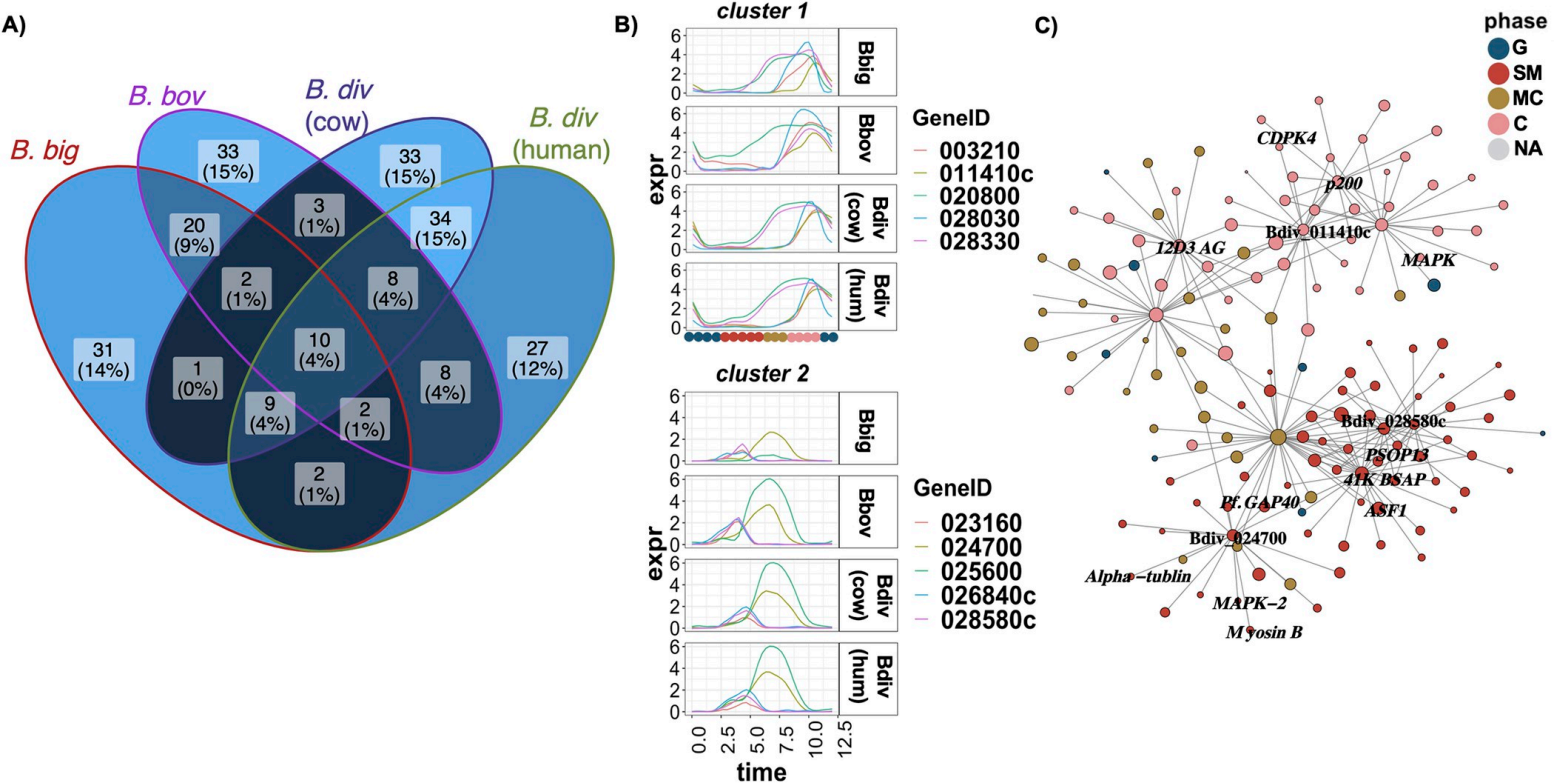
Co-expression network. (A) Figure showing the number of overlapping genes in the indicated contrast. (B) The expression curves of the 10 conserved hub genes at the intersection grouped into 2 clusters with similar expression profile. (C) The inferred interactome of 10 conserved hub genes in the *B*. *divergens* (human) co-expression network. NA, not available. Data and code for generating the figure are available at https://github.com/umbibio/scBabesiaAtlases.

The presence of clusters of co-expressed genes (Fig 5) indicates that shared mechanisms may orchestrate the expression of gene clusters and the progression of the replication cycle. To shed light on the interaction between co-expressed genes, we assembled co-expression gene–gene interaction networks using a probabilistic graphical model [71] (Materials and Methods). A gene interaction network is a graph where nodes are genes and edges represent connection between the genes. The connected genes in the network represent genes that are potentially functionally related. Gene interaction networks are extensively used for a variety of applications, such as identifying clusters of functionally related genes or inferring function through association [72]. Highly connected genes in the network (i.e., “hub genes”) correspond to genes that may have an essential function. Disruption of highly connected hubs in interaction networks causes a major shift in the topology of the network [73]. Since the cell cycle is the major source of variation in our data, the hub genes may have a significant role in the progression of the cell cycle. We identified and ranked the top hub genes in each species and analyzed the overlap of their interactomes (i.e., genes connected to the hub-genes) across all species (Fig 7A). There are 10 genes at the intersection of hub genes in all 4 datasets. Of these 10 genes, 3 are annotated as surface antigens—41K blood-stage antigen precursor 41–3 (Bdiv_026840c), 12D3 antigen (Bdiv_020800), and 200 kDa antigen p200 (Bdiv_003210)—and there is also the highly conserved rhoptry-associated protein 1 (*rap-1*, Bdiv_025600). Rap-1 is known to be important in host cell invasion, an essential and conserved process in *Babesia* [34,74–76]. Fig 7B shows the pseudo-time expression of the 10 genes, clustered in 2 groups. Fig 7C shows the *B*. *divergens* (human) sub-network of the 10 genes at the intersection. The network appears to be modular, with most genes in the C or SM phase. Hub nodes in the interactome include *asf-1* (Bdiv_015780c), mitogen activated protein kinase (*mapk*, Bdiv_023270), and calcium-dependent protein kinase 4 (*cdpk4*, Bdiv_024410). ASF-1 has been shown to play an essential role in histone organization and progression through S phase in other systems [77,78]. The MAPK identified shares sequence homology with ERK7 kinase in *T*. *gondii* (TGGT1_233010), which has a role in the stability of the apical complex [79–81]. The hub gene *cdpk4* has been previously shown to be essential in egress [39]. Interestingly, several hypothetical proteins emerge at the intersection of hub genes, including Bdiv_011410c, Bdiv_024700, and Bdiv_028580c. By determining the interactome of these genes, some inferences about their cellular function can be made.

The hypothetical protein Bdiv_028580c shares the majority of its connections with genes in the SM phase (20 of 36 connections). Interestingly, it is connected to an aspartyl protease (*asp6*, Bdiv_022420c) that shares sequence homology with plasmepsin VII of *P*. *falciparum*. In *P*. *falciparum*, this aspartyl protease plays an essential role in the invasion of the mosquito midgut in the ookinete stage [82]. In addition to this aspartyl protease, this hypothetical protein is also connected to Bdiv_010620, which is orthologous to the secreted ookinete protein *psop13* in *B*. *microti*, and the orthologous gene in *Plasmodium* (PF3D7_0518800) is known to play an important role in transmission [83]. Finally, this hypothetical protein is connected to the histone chaperone FACT-L (Bdiv_010540c), which is known to play a key role in male gametocyte development in *P*. *berghei* [84]. FACT-L expression occurs during DNA replication in *P*. *falciparum*, which is consistent with our finding of this hub gene occurring in the inferred SM phase [24,26]. Taken together, these observations suggest a role for Bdiv_028580c in pre-sexual development and suggest this process may be initiated in blood-stage *Babesia* parasites. Given the lack of an obvious sexual stage in the presented single-cell data (Fig I in S1 Text), this suggests that parasites may express sexual-stage genes in blood-stage development to be primed for possible transmission stimuli should they occur. Alternatively, the expression and connection of these markers mainly in the SM inferred phase may indicate an important yet unknown function for these genes in the asexual replicative cycle. Previously, other genes known to be important for *Plasmodium* sporozoite and ookinete development have been found to be expressed in the asexual blood stage of *B*. *divergens*, for example *celTOS* (Bdiv_028030), further showing that canonically sexual-stage genes are expressed in the replicative cycle [39]. Further, genes such as *ama1* have been shown to be important in invasion in multiple *Plasmodium* life cycle stages [85,86].

The hypothetical protein Bdiv_024700 shares some homology with *ron6* in *T*. *gondii* (23% identity, TGGT1_297960B), suggesting a possible role in invasion. Bdiv_024700 has several connections to genes important to cytoskeletal arrangement, daughter cell formation, and egress, suggesting a role for the gene in cytokinesis and egress, and most connections occur between the SM and MC phase. One such connection is to *mapk-2* (Bdiv_027570c), which is essential in initiation of mitosis and daughter cell budding in *T*. *gondii* [87]. This hub is also connected to the integral cytoskeletal components myosin B (Bdiv_024680) and α-tubulin (Bdiv_038490), as well as Bdiv_020490, which shares sequence homology with glideosome-associated protein 50 (GAP50, PF3D7_0918000) in *P*. *falciparum*, which plays a role in the organization of the inner membrane complex [88]. Indeed, similar cytoskeletal components are required for invasion in *T*. *gondii* [89]. Furthermore, Bdiv_024700 is connected to an aspartyl protease (*asp2*, Bdiv_023140c) similar to plasmepsin IX and X in *P*. *falciparum*, and has a known role in invasion [39]. Together, these data suggest a role in the invasion process for Bdiv_024700.

Finally, looking at the interactome of Bdiv_011410c, most gene connections occur in the inferred C phase. This hypothetical protein is connected to many genes involved in signal transduction, most notably 2 calcium-dependent protein kinases (*cdpk4*, Bdiv_024410; protein kinase domain containing protein, Bdiv_033990c) and the aspartyl protease *asp3* (Bdiv_006490c), suggesting a possible role in signaling processes that control egress [39]. Taken together, the network analysis identifies genes with essential function in the cell cycle as hubs of the network and enables inference of the function of unknown genes through analysis of the function of neighboring genes.

### Expression profiles of functionally related gene families

**Fig 8:**
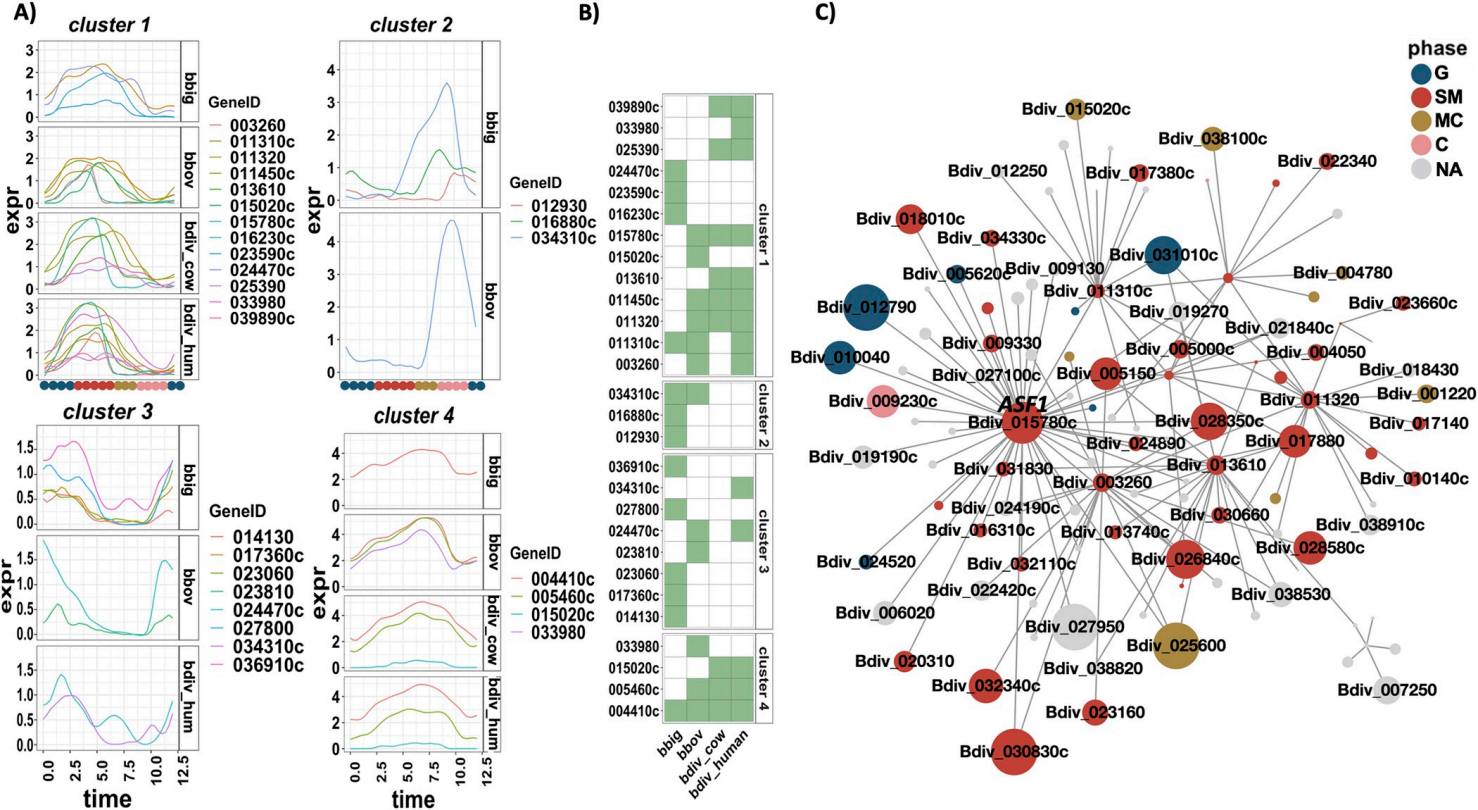
Expression profile of epigenetic markers with a cyclic pattern of expression. (A) The expression curves of epigenetic marker genes clustered into 4 groups according to their expression similarity, split by species. (B) Presence (green) or absence (white) of the gene (rows) in the indicated sample (column); absence indicates that the gene is either not cyclically expressed or belongs to a different cluster (033980, 024470c, 015020c, and 034310c). (C) Interaction co-expression network of epigenetic marker genes in *B*. *divergens* (human) in cluster 1 (9 total). Data and code for generating the figure are available at https://github.com/umbibio/scBabesiaAtlases.

To examine the expression changes in functionally related genes, we performed a clustering analysis on a list of curated gene families: (1) putative transcription factors (TFs) identified by the presence of a DNA binding domain in the sequence, (2) putative AP2 TFs identified by orthology with *P*. *falciparum*, (3) putative epigenetic factors similarly identified by orthology with *P*. *falciparum*, and (4) variable erythrocyte surface antigen (VESA) genes (S5 Table). The reconstructed expression curves of each gene family in each species were clustered into 4 groups, selected to match the 4 inferred phases of the cell division cycle. Fig 8A shows the clustering of epigenetic markers with the cyclic expression profile in all species. Four clear clusters emerge, with many genes peaking at similar times across all species, although the expression patterns of some genes are species-specific. Many of the core histone proteins are co-expressed in cluster 1, showing timing of expression mainly during the inferred SM phase. This pattern of coordinated expression of core histone proteins has been observed in related parasites [90]. There is also a coordinated expression in the MC phase in cluster 3, including epigenetic factors involved in chromatin organization (Bdiv_023810, Bdiv_024470c, Bdiv_036910c) and histone modification (Bdiv_023060, Bdiv_034310c). Cluster 2 shows peak gene expression of 3 epigenetic genes that occur in C phase, a histone demethylase (Bdiv_012930), histone acetyltransferase (Bdiv_034310c), and a zinc-finger domain containing protein (Bdiv_016880c). Cluster 4 shows broad expression over the replication cycle, and the genes that compose this cluster do not fall into similar gene families. Fig 8B shows a heatmap representing the presence of each cyclical epigenetic marker in each cluster and species. Absent genes in a species either are not cyclically expressed or belong to a different cluster. We next identified the inferred interactome of these markers using the co-expression networks. Fig 8C shows the interaction network of epigenetic markers in cluster 1 for *B*. *divergens*. Interestingly, many of these genes appear as hubs in the network including *asf-1*, which is the top hub gene in *B*. *divergens* in bovine blood and among the top in *B*. *divergens* human blood and appears in the inferred interactome of 10 conserved hubs (Fig 7C). ASF-1 shares connections with histone H2A (Bdiv_011310c), histone 2B (Bdiv_011450c), and chromatin assembly factor 1 (*caf-1*) subunit C (Bdiv_013610), each of these occurring in cluster 1 (Fig 8A). Identifying this interaction provides support for the validity of the networks generated from these data in *Babesia* spp.

The same analysis was performed for TFs with cyclical expression profiles, including AP2 domain containing proteins, identified through reciprocal blast with identified TFs in *P*. *falciparum* [91] (Fig J in S1 Text). We identified 4 clusters of co-expressed genes, which map to the 4 inferred replication cycle phases, with each gene identified by its ID in *B*. *divergens* (Fig J in S1 Text). Several AP2 domain containing proteins appear to show a cyclical expression profile in 1 species. For example, Bdiv_037050 (AP2-G3) peaks at G phase in *B*. *divergens*, and Bdiv_010110c peaks during SM in *B*. *bigemina*. In contrast, the AP2 domain containing proteins Bdiv_000800c and Bdiv_024900c are expressed at the same time in all species. There appears to be a highly conserved function in the inferred SM phase for Bdiv_024900c—an AP2 domain containing protein with sequence homology to PF3D7_1239200. The expression of this gene is earlier than in the expression profile of *P*. *falciparum*, where it peaks in the later stages of schizogony [92]. The most similar ortholog of this gene in *T*. *gondii* is *AP2VIIb-3* (TGGT1_255220), which is implicated in the replication cycle progression into S phase, which is more in line with the observed expression across *Babesia* species [93,94]. A list of gene cluster IDs is available in S6 Table.

### Motif analysis of the interactome of TFs and AP2

**Fig 9:**
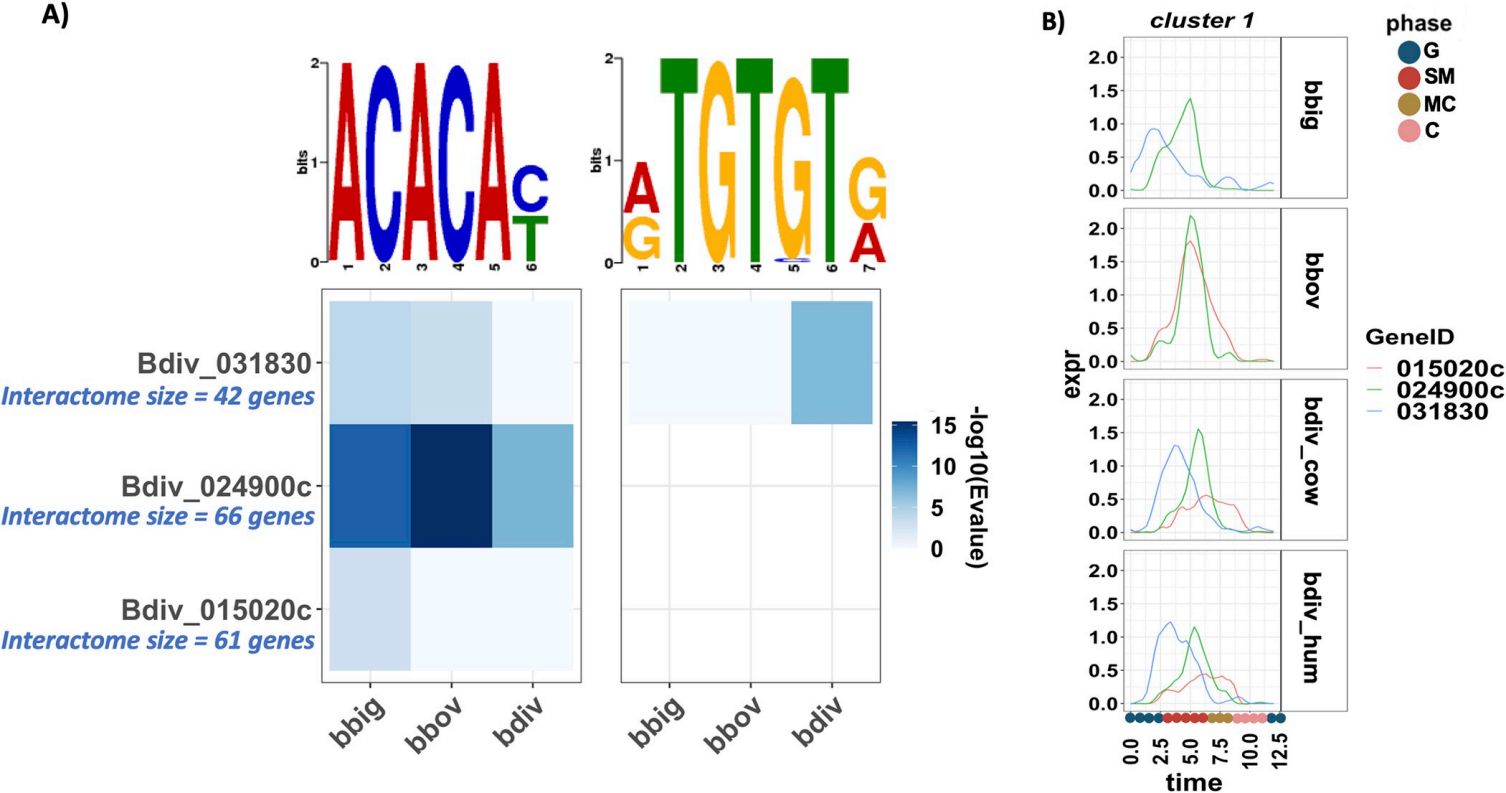
Motif search analysis identified a significant motif in the promoter of the interactome of 3 AP2s. (A) The heatmap shows the significance (−log10(E-value)) of identified motifs, with rows corresponding to the interactome of the indicated AP2 and columns corresponding to the species. (B) Expression curve of the 3 AP2s. Data and code for generating the figure are available at https://github.com/umbibio/scBabesiaAtlases.

To examine whether the interactome of the TF and AP2 family of regulators is transcriptionally co-regulated, we performed a motif search analysis using MEME suite [95] on the promoter genes in the interactome. For each AP2 (and TF), we identified the genes interacting with the AP2 as determined by the assembled co-expression network. Next, we took the union of all genes across the 3 species to assemble a shared interactome for each AP2. The promoter sequences of the genes in the interactome of each AP2 were extracted for each species independently and inputted into the MEME algorithm. The analysis identified a significant motif “ACACA” in the promoter of the interactome of 3 of the AP2s: Bdiv_015020c, Bdiv_024900c, and Bdiv_031830 (Fig 9A). These AP2s are orthologous to *P*. *falciparum* AP2s PF3D7_0604100 (SIP2), PF3D7_1239200 (an unstudied AP2), and PF3D7_0802100 (AP2-I), respectively. Interestingly, the motif “ACACA” has previously been reported to play a role in DNA replication in *Plasmodium* spp. [96,97]. Moreover, analysis of ATAC-seq regions in *P*. *falciparum* has identified and associated the same motif with AP2-I (PF3D7_0802100) [98]. Fig 9B shows the peak time expression of the 3 AP2s in all species. The AP2s mostly cluster together and seem to peak at S/M phase.

### Interactive web app

**Fig 10:**
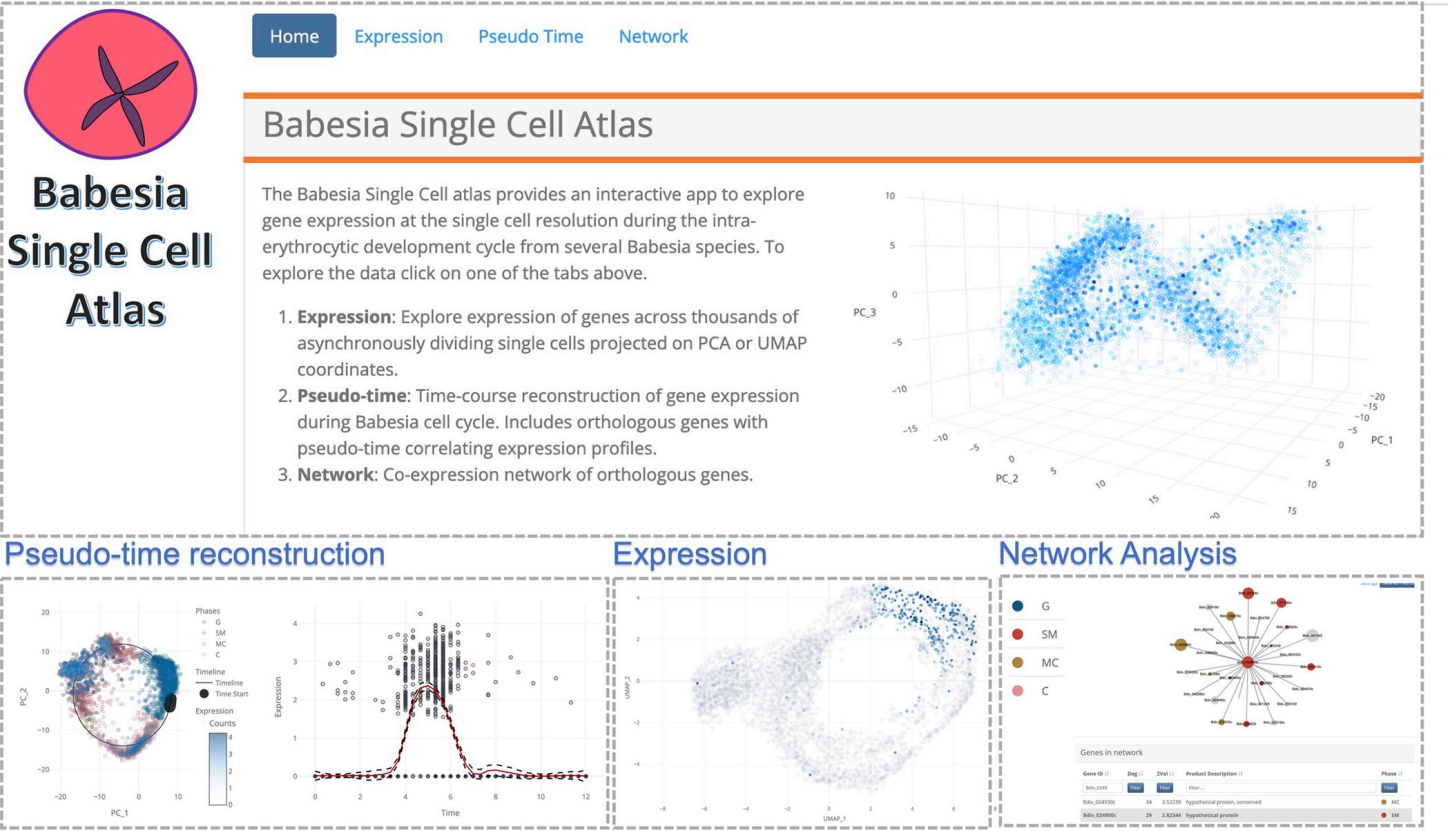
*Babesia* single-cell atlas screenshots. The app provides functionality for pseudo-time analysis, visualizing expression, network analysis, and comparative transcriptomics. URL: https://umbibio.math.umb.edu/babesiasc/. Data and code for generating the figure are available at https://github.com/umbibio/scBabesiaAtlases.

To facilitate usage, we developed a user-friendly interactive web app using web dashboard. The app provides functionality to explore and visualize gene expression during the IDC across thousands of asynchronously dividing single cells projected on PCA or UMAP coordinates. Users can examine the timing of expression using pseudo-time analysis and perform comparative transcriptomic analysis across the *Babesia* species. Moreover, users can generate co-expression networks and interactively visualize and explore the inferred interactome of genes. Fig 10 illustrates the main functions implemented in the app. The app can be accessed at https://umbibio.math.umb.edu/babesiasc/. The source code for the app is available on GitHub at https://github.com/umbibio/babesia_dash_app.

## Discussion

In this work, we present the first single-cell sequencing data in asynchronously replicating *Babesia* parasites and characterize the progression of the replication cycle using newly developed computational approaches. The replication cycle in *Babesia* spp. likely relies heavily on various signaling pathways (reviewed in [28]). In the asexual replicative cycle of *B*. *bovis* and *B*. *bigemina* and many other species, after invasion, the vast majority of parasites will grow, mature, and divide once to form 2 daughter cells prior to egress; for other species, parasites will divide into 4 daughter cells [99]. Data on the division cycle for *B*. *bovis* in asynchronous culture suggests that cell division is complete in approximately 5 h, yet this does not describe the time from invasion to egress [100]. However, in a more recent study on synchronized *B*. *bovis*, this process was approximately 12 h [38]. In contrast, *B*. *divergens* has a much more complex replicative cycle [37]. Indeed, conflicting literature exists suggesting that the replication cycle varies from 4 to 12 h; however, in most cases it appears the minimal time for division is between 4 and 5 h [37,101,102]. Of these studies, only one was conducted on synchronous parasites [37]. However, data also show that the time for the majority of parasites in *B*. *divergens* to transition from single parasites to paired piriforms is between 10 and 12 h [37]. Subsequent division cycles are possible in *B*. *divergens*, and the timing of these cycles can vary between 9 and 14 h [37]. In all studies of the dynamics of *B*. *divergens* division, there is a significant range of time for a single division, highlighting the difficulty in generating an exact measurement [37,101,102]. This variability leads to difficulty in synchronizing *Babesia* parasites, which currently relies on mechanical release of parasites from RBCs using filtration [37,38], and we have recently shown that parasites may be egress competent at various times in their intraerythrocytic development [39]. Unfortunately, no such detailed data on the division time in *B*. *bigemina* exist; however, the replication rate in culture is similar to that in *B*. *bovis*, and the 2 parasites follow a similar pattern of dividing from single to double parasites prior to egressing. Based on these data, we opted to set the window of time for the replication cycle at 12 h for the 3 parasites [39]. Of note, in our analysis we did not distinguish separate clusters of expression for secondary division cycles in *B*. *divergens*, suggesting that the core replication process is conserved and that multiple rounds of replication within the same RBC do not require separate gene regulators.

Taking advantage of the unique geometry of replicating parasites, we developed a pseudo-time analysis and used the synchronous bulk RNA-seq data in *B*. *divergens* to calibrate the progression of time in single-cell data. This technique allows us to generate pseudo-synchronized time-course data at a fine resolution and reconstruct the expression waves of genes during the replication cycle. Analysis of the data shows a high degree of agreement between the bulk and single-cell data, demonstrating the ability of single-cell measurements to match (and overcome some of the limitations of) synchronized time-course measurements.

The limitations, challenges, and advantages of scRNA-seq have been extensively reviewed, including comparisons of the most robust tools, understanding dropouts, and discussion of the ability to understand genes with low expression [103–110]. While scRNA-seq offers extremely fine granularity of cell states, the resolution with which differential gene expression can be detected varies significantly [109]. Indeed, in these *Babesia* spp. datasets, we can clearly observe divergence between bulk RNA-seq and scRNA-seq in the pattern of expression over time in genes with low expression. For example, protein kinase G (*PKG*, Bdiv_020500) was recently identified as an essential gene in egress using synchronized bulk RNA-seq and reverse genetics [39]; however, this gene has low expression, and the single-cell data have difficulty detecting this gene (Fig K in S1 Text). This highlights a key challenge to all methods of differential gene expression: There is often not enough power to confidently characterize genes with low expression [109]. In mammalian cells, it is recommended that one sequence to a depth of 20,000 reads per cell. Based on the minimal *Babesia* genome size and decreased number of predicted coding sequences (between 3,700 and 4,000) in relation to mammalian cells (>12,000), we estimated that an average of approximately 6,000 reads per cell should sufficiently capture the expression profiles (S1 Table). We acknowledge that this depth is lower than that of scRNA-seq experiments performed in *Plasmodium* and *Toxoplasma*; however, *Babesia* has both a smaller genome and fewer coding sequences predicted than these related organisms (3,700 compared to approximately 5,200 and 8,100 for *Plasmodium* and *Toxoplasma*, respectively). We also note that the read depths do vary between samples (correlating with genome size) and may cause some limitations in the downstream data analysis of genes with low expression. These limitations are important to consider when attempting to characterize cell populations, especially those that may represent rare cell types.

However, scRNA-seq also provides major advantages over bulk sequencing techniques. In most synchronized bulk RNA-seq time-course studies, measurements are limited to a few discrete time points (typically <12 points), whereas in sequencing heterogenous single-cell populations, the entire trajectory of expression can be captured, representing a continuum of time. This is advantageous in capturing subtle variations in curvature, which allow more precise mapping of peak expression as well as clustering genes by expression similarities. Because of the ability to capture time using asynchronous populations, the difficulties of tightly synchronizing parasites are completely avoided in single-cell studies. Indeed, heterogeneity is an advantage in scRNA-seq that allows capturing and grouping cells in similar states at single-cell resolution, and scRNA-seq can capture and characterize small cell populations not distinguishable at the bulk level. In this study, we highlight the benefit of combining the 2 techniques to leverage the detection power of bulk RNA-seq and the fine resolution of single-cell sequencing.

It should be noted that annotations of *Babesia* genomes are not extensive and do not include the untranslated regions (UTRs). Moreover, the gaps between the genes are shorter compared to *T*. *gondii* and *Plasmodium* spp. (S2 Table). However, there is generally a clear boundary between mapped reads to the genome, and intergenic regions (including unannotated UTRs) are excluded in calculating transcript abundance. While it would be optimal to include UTRs, the reads mapped to the exons provide a good approximation for transcript abundance, and there is no bias toward any specific gene when calculating relative abundance and DEGs.

Using a comparative approach, we utilized data from better studied *T*. *gondii—*where the replicative cycle phases have been characterized using single-cell sequencing and DNA content [50]—to map the replication cycle phases in *Babesia* spp. and identify the DEGs of “inferred” replication cycle phases. The significant overlap between peak expression times of *T*. *gondii*–based markers indicates that progression of cell division in *Babesia* spp. may not fit well into the canonical model of cell cycle progression (Fig D in S1 Text). Mapping transition points in the *Babesia* replication cycle can also be achieved using automatic clustering approaches. We initially used this approach and identified 5–6 phases, which mostly agree with *T*. *gondii* inferred phases, with little impact on GOEA results (Fig C in S1 Text). The progression of gene expression (Fig 5) shows a defined transition point between G and S/M phases, and a gradual shift through S/M/C with overlapping transition boundaries. More data, (e.g., time-course DNA content) is needed to map the phases more accurately. However, GO indicates that the inferred phases of canonical *T*. *gondii*–based phases agree with the known biology of the replication cycle.

The mapping of replication cycle phases allowed us to identify phase-regulated genes and perform comparative studies between the *Babesia* species. The analysis revealed conserved and species-specific genes that delineate the inferred states of the replication cycle. Intriguingly, although several of the genes have species-specific expression profiles, the total number of DEGs in each phase is comparable, and the progression of gene expression during the replication cycle shows a similar pattern in all species. We also investigated the impact of the host species on the transcriptome. Of the parasites tested, *B*. *divergens* is able to grow in both human and bovine RBCs, while *B*. *bigemina* and *B*. *bovis* can only be propagated in bovine RBCs. The genes that were identified with differential gene expression in *B*. *divergens* between bovine and human RBCs indicated changes in the expression of genes involved in transcriptional regulation, as well as those potentially involved in invasion and egress. We also identified several differentially regulated aspartyl proteases known to play roles in invasion and egress in various replication cycle phases [39]. The differential expression of these genes could be due to differences in host cell receptors, parasite ligands, or host cell membrane composition. A common thread through the DEGs between human- and bovine-adapted parasites is the presence of changes in protein metabolism, signaling, and vesicular transport. Despite both being mammalian hosts, bovine and human RBCs differ in their composition, size, and deformability [111–114]. One striking difference between bovine and human RBCs is the lipid composition of the cell membrane. The composition of bovine RBCs is significantly divergent from most other mammals in that they have low to absent levels of phosphatidylcholine, while having high sphingomyelin levels [112]. This stark difference in lipid composition of the host cell membrane may be a driver of the differential expression; indeed we did observe upregulation of 2 genes involved in lipid metabolism in parasites resident in human RBCs (Bdiv_036170c, Bdiv_025380c). Lipid composition can also affect signal transduction [115], which could underlie the differences we observed in the expression of genes involved in protein kinase activity. Future experiments comparing these processes between bovine- and human-adapted parasites are warranted based on the differences observed here.

Interestingly, the major unifying difference in bovine parasites is the emphasis on vesicular transport and the endomembrane system. One hypothesis from this observation is that parasites grown in bovine RBCs require increased trafficking to and from the membrane, both to export proteins and scavenge nutrients. The ability of *B*. *bovis* to cause disease pathology by adhering to the vascular endothelium via altering the RBC membrane surface supports the idea that increased protein export may occur in bovine parasites [116–120]. Recently, expression of a multigene family of multi-transmembrane integral membrane proteins (*mtm*) was identified in the proteome of the infected RBC membrane, in addition to several other multigene families (*ves1*, *smorf*, *tpr-*related), showing this protein is expressed and exported by the parasite [121]. These processes in *B*. *bovis* could account for an increased use of the endomembrane system. However, this cannot fully explain the observed enrichment across the bovine-adapted parasites, as *B*. *bigemina* and *B*. *divergens* do not sequester. It may still be the case that parasites in bovine RBCs more dramatically alter the host cell. Indeed, when splenectomized calves were infected with stabilates of bovine-derived strains of *B*. *divergens*, an increase in the mean corpuscular volume of the host cell was observed in relation to infection by human-derived patient isolates (stabilates), suggesting changes in the membrane architecture. This suggests there is a parasite-specific effect on bovine host cells depending on which host (human versus cow) the parasite was originally isolated from [122]. Unfortunately, no such studies investigating protein export or RBC architecture exist as of yet in *B*. *bigemina* [120]. However, the evidence from *B*. *bovis* and *B*. *divergens*, combined with the pattern observed in this study, presents an intriguing possibility that protein export is affected by resident host cell.

Our work provides to our knowledge the first comparative single-cell transcriptomic study across 3 *Babesia* species. To date, comparative genomic studies have focused on gene annotation, understanding variant gene expression, and elucidation of virulence determinants [17,18,20,29,31–33,35,36,123]. In the context of this study, it is worth noting that the organization of the genomes of *B*. *bovis* and *B*. *divergens* share similarity [18]. Additionally, *B*. *bigemina* contains extensive duplications of certain gene families, leading to an increased genome size [32]. The genome sequences would suggest that similarities exist in general cellular development, and variations arise due to host evasion pathways and differential host tropism. Several comparative studies have sought to understand the expression and structure of VESAs, both within and across species [32,36,123]. Consistent with these studies, we also observed a sporadic expression pattern of VESA genes, with species-specific differences (Fig L in S1 Text).

We also utilized our dataset to examine the expression profile of other gene families, including, AP2s, TFs, and epigenetic markers [92,124]. The analysis revealed distinct clusters of genes with similar peak expression time across species, indicating that shared regulatory mechanisms may be orchestrating the progression of the replication cycle. Of note, we identified that several core histones are co-expressed, which is also observed in related parasites [91]. Interestingly, the timing of expression of these core histone proteins differs from that in related parasites: For example, histone H3 expression peaks in early schizonts towards the end of DNA replication in *P*. *falciparum* [25], while in the 3 *Babesia* species the expression peaks around 3.75 h, which is at the beginning of S phase. This difference is likely indicative of the different modes of division of the parasites (schizogony versus binary fission). We were also able to provide strong evidence for the utility of the interactome networks by showing the connection of *asf-1* to histone expression (Fig 8C). Interestingly, *asf-1* appeared throughout our analyses, suggesting an important role of the gene in *Babesia* asexual cycle development. Future studies disrupting *asf-1* in *Babesia* would provide insight into the nature of the regulation of chromatin formation, as well as reveal any novel functions for the gene in the parasite. These interactomes provide the foundation for future perturbation experiments to understand the directionality of regulatory interactions. Additionally, we were able to identify patterns of expression of AP2 domain containing proteins that were dependent on replication cycle phase (Fig J in S1 Text) and were able to identify a conserved motif for a set of these TFs (Fig 9B).

Finally, to facilitate wider use, we present a web dashboard for interactive exploration of the data. The app provides functionality for analysis of the expression of single genes, comparative analysis of expression profiles and pseudo-time curves across all species, and co-expression network analysis of genes. The app is hosted at https://umbibio.math.umb.edu/babesiasc/. This resource will allow for novel studies to expand upon and add to the analyses of these rich transcriptomic datasets using the interactive interface, without the need for expertise in computational methods. The work described here lays the foundation for numerous functional studies to elucidate many facets of *Babesia* biology.

## Materials and methods

### Parasite culture

The *B*. *bovis* strain MO7 and the *B*. *bigemina* strain J29, provided by David Allred of the University of Florida, were maintained in purified bovine RBCs (hemostat) hematocrit in RPMI-1640 medium supplemented with 25 mM HEPES, 11.50 mg/l hypoxanthine, 2.42 mM sodium bicarbonate, and 4.31 mg/ml AlbuMAX II (Invitrogen). Before addition of AlbuMAX II and sodium bicarbonate, we adjusted the pH of the medium to 6.75. *B*. *divergens* strain Rouen 1987, kindly provided by Kirk Deitsch and Laura Kirkman (Weill Cornell Medical College), was maintained under the same conditions in purified white male O+ human RBCs (Research Blood Components). All cultures were maintained at 37°C in a hypoxic environment (1% O_2_, 5% CO_2_). Clonal lines of parasites were used for all selections and were derived from the provided strains via limiting dilution—these will be referenced as BdC9 (*B*. *divergens*), BigE1 (*B*. *bigemina*), and BOV2C (*B*. *bovis*).

### Single-cell protocol

*B*. *divergens*, *B*. *bovis*, and *B*. *bigemina* were grown to >15% parasitemia. Health of parasites was assessed by thin blood smear. Parasites were collected and pelleted, and washed with warm 1× PBS, followed by a final wash with warm 0.4% BSA in 1× PBS. To ensure loading of the correct number of parasites, parasitemia was counted on stained thin blood smears (2,000 total cells), and RBCs were counted using a hemocytometer. These values were used to calculate the number of infected cells to be loaded into the Chromium Chip B. We aimed for recovery of 10,000 infected cells, thus loaded 16,500 infected cells in bulk culture. Cell suspensions were loaded into individual wells on the Chromium Chip B. After gel bead-in-emulsion (GEM) generation, single-cell libraries were processed according to the 10× Genomics Chromium 3′ v2 User Guide protocol, using 13 amplification cycles for cDNA amplification, and 14 cycles in library construction. Libraries were subsequently sequenced on the Illumina NextSeq platform following the 10× Genomics specifications, aiming for a minimum of 6,000 reads per cell.

### RNA-seq alignment

The reference genomes and annotation files of *Babesia* spp. (release 54) were downloaded from PiroplasmaDB (https://piroplasmadb.org/). Custom references were generated using the 10× Genomics Cell Ranger (version 6.0.0) pipeline (cellranger mkref), and raw fastq files were aligned to the genome cellranger count with default parameters.

### scRNA-seq data processing

All data analysis was performed in R (version 4.1.1). The R Seurat package version 3 [53] was utilized to process the count data. Seurat objects were created for each count independently. Cells and genes with low counts were filtered out from the analysis using the Seurat function CreateSeuratObject with parameters min.cells = 10, and min.features = 100. Expression data were normalized using the Seurat functions FindVariableFeatures (with parameter nfeatures = 3,000) and ScaleData. Dimensionality reduction was performed using PCA and UMAP as implemented in the Seurat functions RunPCA, and FindNeighbors, with parameters dims = 1:10 and reduction = pca. Clustering analysis was performed using the *k-*nearest neighbors algorithm using Seurat function FindClusters with parameter res = 0.2. Datasets were down-sampled to include 800 cells per cluster. Orthologous genes in all 3 species were used to construct Seurat objects with same genes using *B*. *divergens* gene IDs. Datasets were then integrated using Seurat’s merge and IntegrateData functions. The *B*. *divergens* sample in human host RBCs was used as the “reference” dataset for integration (Seurat FindIntegrationAnchors function).

### Pseudo-time analysis

Pseudo-time analysis was performed in 3 steps. First, an ellipsoid was fitted to the first 2 PCA coordinates in each dataset using the Ellipsefit function from the MyEllipsefit R package (https://github.com/MarkusLoew/MyEllipsefit). Next, the elliptic fit was used as prior to fit a principal curve to each dataset [54]. The function principal_curve from the R package princurve was used to fit the principal curves. The parameter smoother = “periodic_lowess” was set to enforce closed curves. Data were then orthogonally projected onto the principal curves and ordered to generate pseudo-time. The pseudo-time curve was mapped to interval [0,12 h] to mimic the *Babesia* spp. replication cycle. The pseudo-time interval was partitioned into 20-min bins, and cells that fell within the same bin were treated as synchronized replicates. Next, we calculated the correlation of gene expression with pseudo-time using a GAM and filtered out genes that did not correlate with the pseudo-time (FDR-adjusted *p*-value < 0.05). The R function gam from the package gam was used for this analysis. This resulted in a time-course expression matrix with dimensions $n\times \prod_{k=1}^{N}n_{k}$ with *n* representing the total number of genes, *N* representing the total number of time bins (36 intervals, each 20 min), and *n*_*k*_ representing the total number of cells mapping to the time bin *k*.

### Fitting gene curves

We fitted the expression curves with smoothing splines using R’s “smooth.spline” function with the smoothing parameter set by cross-validation. The fitted splines were then sampled at regular time points to interpolate the curves.

### Alignment with bulk RNA-seq data

Time-course bulk RNA-seq data from synchronized *B*. *divergens* parasites was processed as previously described [37]. The bulk time-course data consisted of 7 measurements of synchronized *B*. *divergens* parasites over the 12-h period spanning the replication cycle. There was a total of 2 biological replicates and 2 technical replicates. Technical replicates were merged for this analysis. Smoothing splines were fitted to the common genes between the bulk time-course data and single-cell pseudo-time-course data. The smoothing spline fits to the bulk data were sampled to 36 points, matching the total number of points in the single-cell gene curve data. Cross-correlation between corresponding genes in bulk and *B*. *divergens* single-cell datasets was calculated by

$$g_{b}⋆g_{sc}(t)=\sum_{\tau =0}^{N}g_{b}(t)g_{sc}({\left(\tau +t\right)}_{mod\;N})$$

where *g*_*b*_(*t*) and *g*_sc_(*t*) are gene curves in the bulk and singe-cell data, respectively, and *N* is the total number of sample points in the gene curves. The lag time maximizing the cross-correlation was then calculated for each gene, and the distribution of lag times across all genes was examined to identify a single optimal lag time for all genes. Single-cell gene curves in all single-cell datasets were shifted by the optimal lag time to adjust the start time.

### Inferred replication cycle phases

*T*. *gondii* scRNA-seq data were obtained from the single-cell atlas of *T*. *gondii* [50], where replication cycle phases were determined using DNA content and computational analysis. Markers of each phase were determined by performing differential expression analysis using the Seurat R function FindAllMarkers with parameters only.pos = TRUE, min.pct = 0. Significance was determined using FC > 2 and adjusted *p*-value < 0.01. The top 20 markers of each phase were then used and mapped to their *Babesia* spp. orthologs. The timing of peak expression of each marker was calculated by examining the local maxima of the fitted pseudo-time gene expression curves in each species. Transition time points between phases were then determined by examining the quantiles of the peak time distributions, and adjusted by visual inspection of the overlap of distributions.

### Differential expression analysis

DEGs of the inferred replication cycle phases were identified as follows. Conserved DEGs of each phase across species were identified using the Seurat function FindConservedMarkers with default parameters. DEGs of each phase were also calculated in each species independently using the FindAllMarkers function from the Seurat R package with parameters only.pos = TRUE. DEGs of each phase unique to a specific species were determined using the same function and by setting an appropriate contrast in cell identities (e.g., *B*. *bigemina* G phase versus *B*. *bovis*, *B*. *divergens* (bovine), and *B*. *divergens* (human) G phase). For these analyses, FC > 2 and adjusted *p*-value < 0.01 were used to determine significance. For host-cell-specific differences, differential expression analysis was performed between *B*. *divergens* in human host and *B*. *divergens* in bovine host as well as between *B*. *divergens* in human host and merged *B*. *bigemina*, *B*. *bovis*, and *B*. *divergens* in bovine host in a phased-matched specific manner. For these analyses, FC > 1.5 and adjusted *p*-value < 0.01 were used to determine significance. Genes differentially expressed between *B*. *divergens* and *B*. *bigemina* as well as between *B*. *divergens* and *B*. *bovis* in bovine RBCs were excluded from this analysis to minimize the confounding effect caused by differences in parasites.

### Enrichment analyses

DEGs were mapped to their orthologs in *T*. *gondii*. GOEA was performed using available GO terms on ToxoDB (https://toxodb.org/), and significant GO terms (Benjamini < 0.1) were determined. The log fold enrichment and log *p*-value were used to visualize the significant GO terms.

### Time-course clustering

Mean gene expression curves of inferred replication cycle genes were used to construct an *n*×*N* time-course matrix, with rows representing genes and columns representing pseudo-time bins. Data were scaled to *z*-scores, and a DTW metric was used to measure the similarity between curves and to perform a hierarchical clustering. For this analysis we used the tsclust function from the R package dtwclust (https://github.com/asardaes/dtwclust) with parameters control = hierarchical_control(method = “complete”), args = tsclust_args(dist = list(window.size = 4L). The total number of clusters was set empirically with trial and error. Genes were ordered according to peak expression time, and cells according to their inferred replication cycle phase (transition points along the pseudo-time course). A heatmap was used to visualize the expression profile of the genes.

### Reconstruction of the gene–gene interaction network

We used a Gaussian graphical model (GGM) to assemble a gene–gene interaction network using the scRNA-seq expression data. The GGM can be used to calculate the partial correlation between gene pairs conditioned on the rest of the genes, and thus it captures pairwise relationships between the nodes in the interaction graph. Partial correlation is then used to assemble a gene–gene interaction network, where genes represent nodes and edges represent a direct interaction between them after accounting for tertiary effects. The objective of the GGM is given by

$$\underset{\Theta }{\max}\;\log(\det\;\Theta )-tr(S\Theta )-\lambda {\left|\left|\Theta \right|\right|}_{1},$$

where *S* and Θ are the empirical covariance and precision matrices and *λ* is the sparsity penalty.

To fit this model, we estimated the empirical covariance matrix *S* using the pseudo-time-course gene expression data as follows. First, the mean trend *μ*_*i*_(*t*_*j*_) of each gene *i* at time point *t*_*j*_ was estimated using the expression of replicate cells that mapped to time partition *i*. This mean trend was removed from the expression of genes to de-trend the data:

$$y_{i}^{j_{l},d}(t_{j})=y_{i}^{j_{l}}(t_{j})-\mu_{i}(t_{j}).$$

The superscript represents (replicate) cell *j*_ℓ_ at time bin *j*. The trended data were used to estimate the empirical covariance matrix *S*. As gene expression is periodic during the replication cycle, and assuming a non-time-varying covariance matrix, the GGM model can be directly applied to the de-trend data to capture direct covariations in gene expression. The Objective function of the GGM was then fitted for a grid of *λ* values ranging from 0.01 to 1.0 with step size 0.01. The R package glassoFast was used for fitting the model (https://github.com/JClavel/glassoFast). The fitted precision matrices were converted to partial correlation matrices *P*, which in turn were converted to network adjacency matrices. The scale-free network property for each network was calculated, and the penalty value that maximized the scale-free property was used to identify the “optimal” network.

### Motif search analysis

Annotated *P*. *falciparum* TFs and AP2s were mapped to their *Babesia* spp. orthologs when available (S4 Table). For each TF, the interactomes were identified in the assembled co-expression networks in each species, and the union of all interacting genes across all species was taken as the TF’s overall interactome. The promoter of genes in the overall interactomes of each TF was defined as the sequence *N* nucleotides upstream of the start codon, where *N* was set to 167 in *B*. *bigemina*, 219 in *B*. *bovis*, and 190 in *B*. *divergens*. These promoter lengths were selected as one-half of the mean gap between consecutive genes in each species, calculated from genome annotation files (S2 Table). The promoters were extracted using the getfasta command from the BEDTools package [125] and the latest version of the genome of each species, downloaded from PiroplasmaDB (https://piroplasmadb.org/). Motif search analysis was carried out on promoter sequences using the MEME suite [95]. The meme command with the parameters -dna -minw 4 -maxw 10 anr -nmotifs 10 -revcomp was used to perform the motif search.

### Data visualization

A web dashboard was built to provide easy access to single-cell expression data and analysis results. Data were preprocessed and loaded as tables to a SQL database. The web interface was implemented using the “dash” python framework, which allows building of dynamic, interactive, data-driven apps. The current instance of the app is running in a single server using docker containers. However, the app design and the stateless approach of the framework allows for easy scalability to support increasing traffic as needed. The app for the dashboard is organized as a python module and separated into a sub-module for each of the pages available. URL requests are processed using an index python script, which loads the appropriate UI layout and backend logic. The python app is served by a Gunicorn WSGI HTTP Server, which allows it to spawn multiple workers for the app. MariaDB is used as the SQL server for the app, with connection pools of size 32 for each python worker, allowing multiple persistent connections to the database. A series of bash and python scripts were written to automate the process of database initialization from the TSV datafiles. As the data are expected to remain static through the running time of the app, SQL tables were created with tight data size allocations to help with performance. SQL indices were created such that queries remain fast. SQL unique indices are used where possible, as a way of ensuring the input data’s integrity. The resulting relational database contains 9 tables for each of the species, holding data and metadata for genes, orthologous genes, single-cell expression experiments, spline fits for pseudo-time analysis, and nodes and edges for each of the interaction networks available. Finally, a docker-compose configuration script was written, which contains all relevant configuration parameters in one place to easily deploy the app+sql server services. The app can be accessed at https://umbibio.math.umb.edu/babesiasc/. The source code for the app is available on GitHub at https://github.com/umbibio/babesia_dash_app.

## Supporting information

S1 Table Quality control. Tab 1 contains the alignment metrics. Tab 2 summarizes the total number of genes and cells that pass the Seurat filters. Tab 3 summarizes the total number of significant genes in the fitted generalized additive model (GAM; adjusted *p*-value < 0.01).
(XLSX)

S2 Table Genomic statistics. Tabs 1 and 2 contain information and pie charts on the total length and percentages of exons, introns, and intergenic regions in *Babesia* species (*B*. *bigemina*, *B*. *bovis*, and *B*. *divergens*). *T*. *gondii* and *P*. *falciparum* are also included for comparison. Tab 3 contains strand-specific as well as the overall percentage of overlapping genes. Tab 4 contains summary statistics (mean and median) of gaps between consecutive genes.
(XLSX)

S3 Table Differential expression. Tabs 1–5 contain the list of differentially expressed genes (DEGs) of the inferred cell cycle phases (FC > 2, adjusted *p*-value < 0.01). Tabs 5 and 6 contain the list of conserved and species-specific DEGs. Tabs 7 and 8 contain the list of *B*. *divergens* and species-independent DEGs in the human versus bovine comparison.
(XLSX)

S4 Table GO terms. Tabs 1–3 contain the list of enriched GO terms for conserved and species-specific DEGs and human versus bovine DEGs.
(XLSX)

S5 Table Functionally related genes. Tabs 1–3 contain the list of orthologous *Babesia* transcription factors (TFs), AP2s, and epigenetic markers. Tabs 4–6 contain the list of VESA genes in *B*. *bovis*, *B*. *bigemina*, and *B*. *divergens*.
(XLSX)

S6 Table Gene clusters. Tabs 1 and 2 contain the list of genes in each cluster for epigenetic markers (Fig 8) and TFs and AP2s (Fig J in S1 Text).
(XLSX)

S1 Text Additional figures. S1 Text also contains extended analysis on species-specific markers and the human versus bovine marker analysis.
(PDF)

## Abbreviations

FC: fold change
DEG: differentially expressed gene
DTW: dynamic time warping
GAM: generalized additive model
GO: Gene Ontology
GOEA: Gene Ontology enrichment analysis
IDC: intraerythrocytic developmental cycle
PCA: principal component analysis
RBC: red blood cell
RNA-seq: RNA sequencing
scRNA-seq: single-cell RNA sequencing
TF: transcription factor
UMAP: Uniform Manifold Approximation and Projection
VESA: variable erythrocyte surface antigen
