# Supplementary material for: Comparative single-cell transcriptional atlases of Babesia species reveal conserved and species-specific expression profiles
Source: PLoS Biol. 2022 Sep 22;20(9):e3001816. doi: 10.1371/journal.pbio.3001816 (PMC9531838; doi:10.1371/journal.pbio.3001816)
Supplement: S1 Text — also contains extended analysis on species-specific markers and the human versus bovine marker analysis. (PDF) [file pbio.3001816.s007.pdf]

Supplementary Information:

Comparative single cell transcriptional atlases of *Babesia* species reveal conserved and species-specific expression profiles

Yasaman Rezvani[1¶], Caroline D Keroack[2¶], Brendan Elsworth[2], Argenis Arriojas[1, 3], Marc-Jan Gubbels[4], Manoj T Duraisingh[2*], Kourosh Zarringhalam[1, 5*]

[1] Department of Mathematics, University of Mass. Boston, Boston, Massachusetts, United States of America

[2] Department of Immunology and Infectious Diseases, Harvard T. H. Chan School of Public Health, Harvard University, Boston, Massachusetts, United States of America

[3] Department of Physics, University of Mass. Boston, Boston, Massachusetts, United States of America

[4] Department of Biology, Boston College, Chestnut Hill, Massachusetts, United States of America

[5] Center for Personalized Cancer Therapy, University of Mass. Boston, Boston, Massachusetts, United States of America

[¶]These authors contributed equally to this work

[*] mduraisi@hsph.harvard.edu (MTJ); *kourosh.zarringhalam@umb.edu (KZ)

Fig A

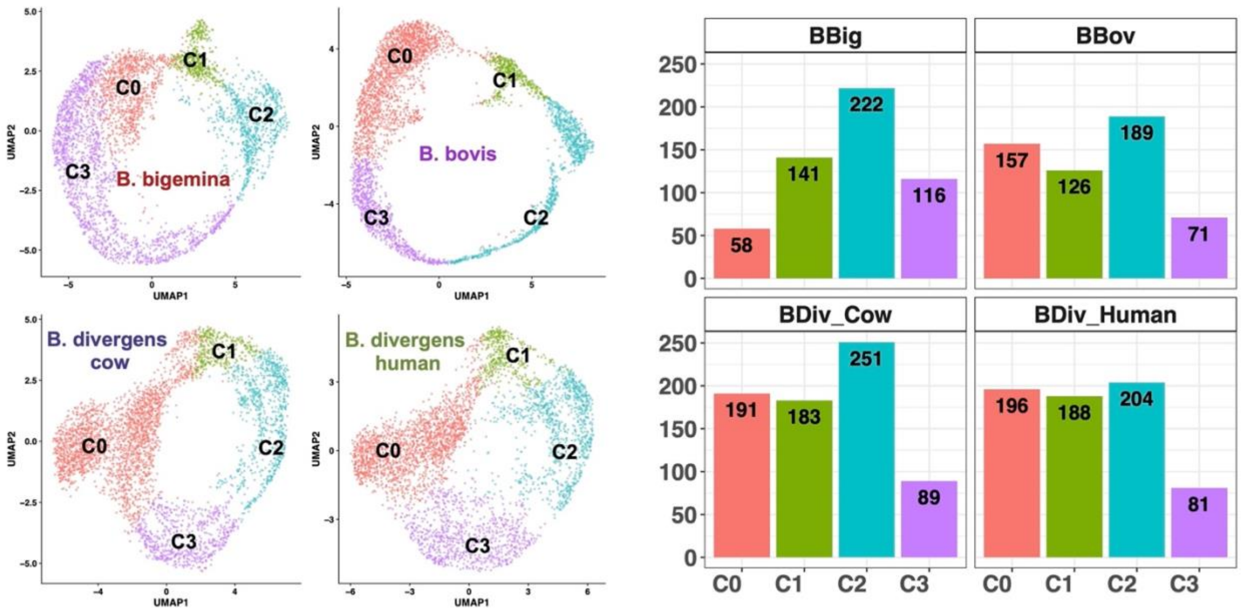

Fig A. (Left) Figure shows individually processed data in all species projected on UMAP coordinates. Colors indicate automatically identified clusters. (**Right)** Markers of each cluster in the indicated species. Data and code for generating the figure is available at https://github.com/umbibio/scBabesiaAtlases.

Fig B

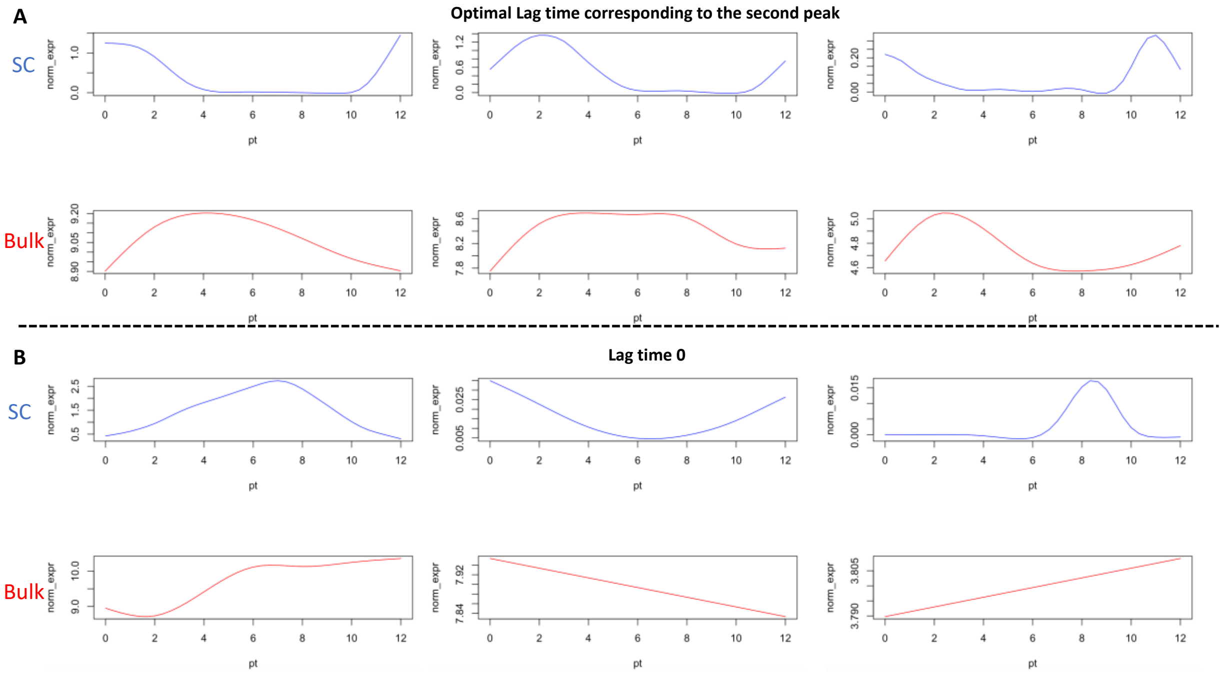

Fig B. (**A**) Examples of gene expression curves for sc (top row; blue) and the corresponding gene in bulk (bottom row; red) whose optimal lag time correspond to the second peak in the lag time distribution (main text **Fig. 2**). (**B**) Similar expression curves for genes whose optimal lag time is 0. These genes primarily consist of monotonically expressed genes. Data and code for generating the figure is available at https://github.com/umbibio/scBabesiaAtlases.

Fig C

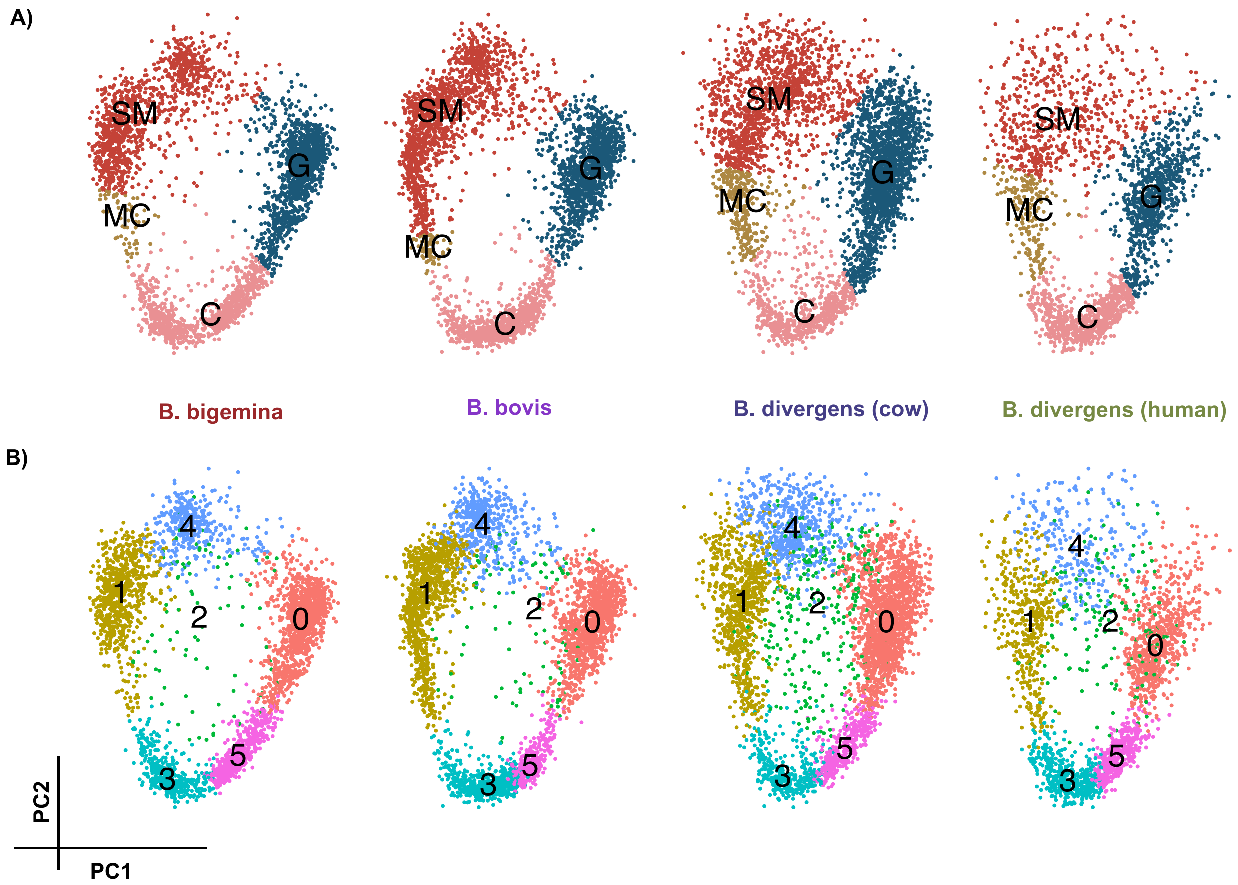

Fig C. Figure shows the projection of all on PCA coordinates. Colors indicate (**A**) inferred cell cycle phases using *T. gondii* markers, and (**B**) cell clusters automatically identified using graph-based clustering. Total number of clusters was set to 6. Data and code for generating the figure is available at https://github.com/umbibio/scBabesiaAtlases.

Fig D

The figure shows few examples of expression the distribution of the peak expression time of the *Babesia spp.* orthologous genes of the top 20 *T. gondii* replicative cycle markers, scaled to 0-12 h. The distribution of markers of the G phase is distinct with low overlap with other phases, whereas markers of S phase and early M phase, as well as M phase and early C phase overlap significantly. As such transition points in canonical S and M as well as M and C phases are not readily discernible by timing of expression alone.

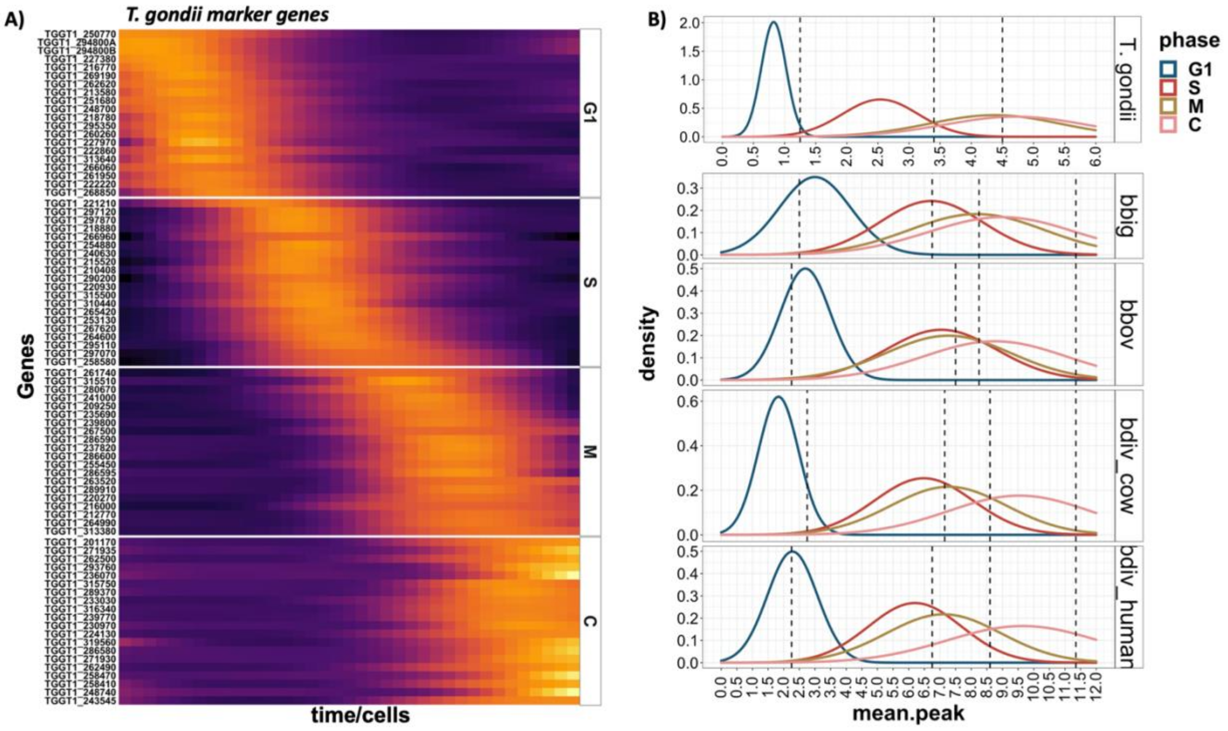

Fig D. (**A**) Heatmap of mean expression curves of top 20 cell cycle phase marker genes in *T. gondii*. Genes with similar expression curve are clustered together. Genes are split according to their identified cell cycle phase from Xue et.al [1]. (**B**) Distribution of the peak time expression of top 20 *T. gondii* cell cycle markers in *T. gondii* (top panel) and all Babesia species. The peak time curves are colored based on indicated cell cycle phase. Vertical dash lines represent the transition time points. Data and code for generating the figure is available at https://github.com/umbibio/scBabesiaAtlases.

Fig E

Figure shows an alternative approach for identifying species specific DEGs. In this approach, species specific DEGs and conserved DEGs were identified by calculating the set difference and intersection of independently identified DEGs across species.

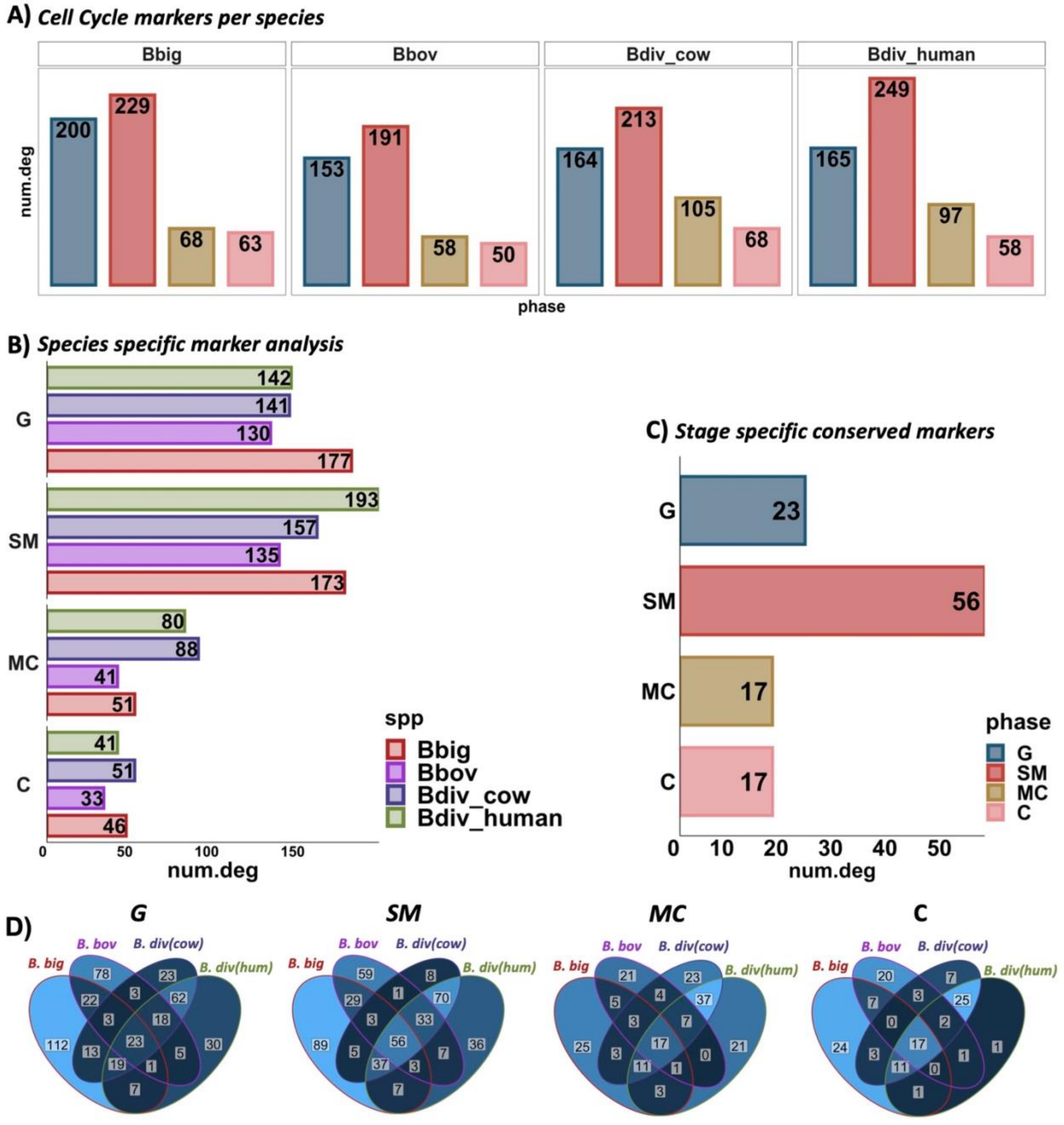

Fig E. **(A)** Total number of inferred replication cycle DEGs in each species independent of other species. **(B)** Total number of DEGs uniquely assigned to indicated species in each inferred

replication cycle. **(C)** Total number of shared DEGs in each inferred phase across all species. For differential expression analysis fold change cutoff > 2 and adjusted p-value < 0.01 were applied to determine significance. **(D)** Venn diagram showing overlap of DEGs across species in each phase. Data and code for generating the figure is available at https://github.com/umbibio/scBabesiaAtlases.

Fig F

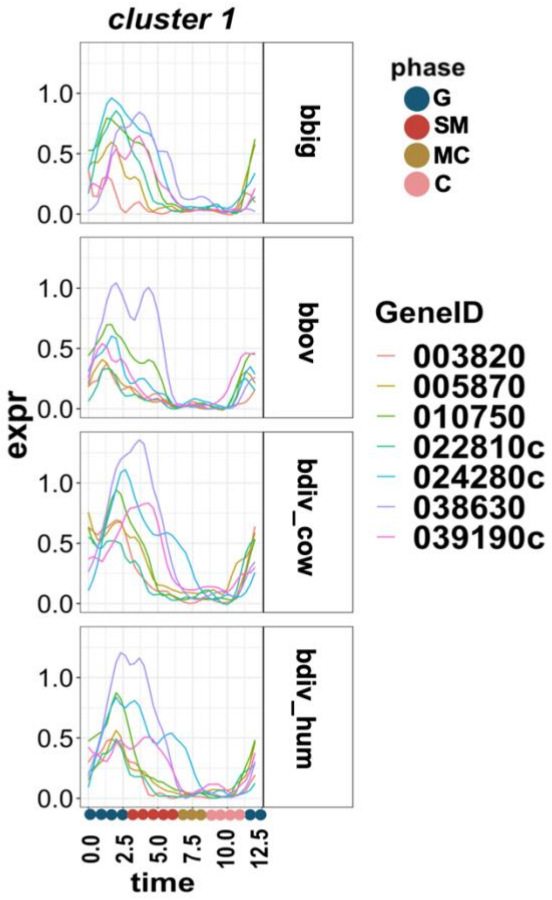

Fig F. Expression profile of genes involved in DNA replication that are markers of inferred G phase, split by species. Data and code for generating the figure is available at https://github.com/umbibio/scBabesiaAtlases.

Fig G

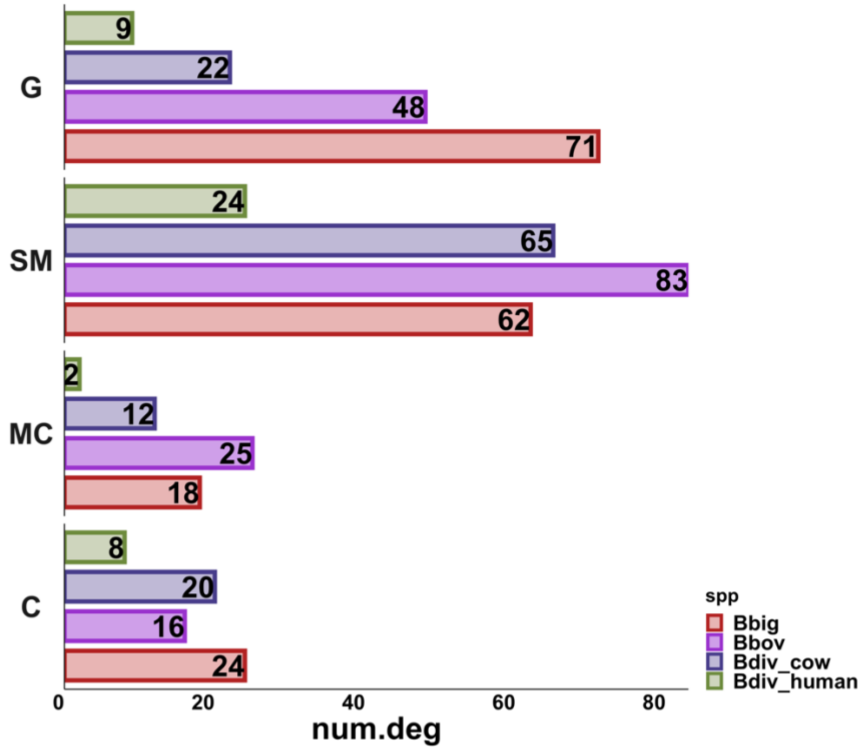

Fig G. Total number of markers uniquely assigned to indicated species (colors) in each inferred replication cycle. For this analysis we used FC > 1.5 and adjusted-p-value < 0.01 to determine significance. Data and code for generating the figure is available at https://github.com/umbibio/scBabesiaAtlases.

Extended analysis of species-specific markers

In addition to conserved markers of replication cycle progression, we also investigated species-specific markers of each inferred state using GO-term enrichment, focusing on the most highly enriched processes. In the inferred G phase of the three parasite species, the main differences occurred in metabolic processes and nutrient scavenging. In *B. bigemina,* there was strong enrichment for various transmembrane transport processes, the most highly ranked being nucleotide (ATP) transport (BBBOND_0211990), known to be important in mitochondrial transport in related parasites [2,3]. In *B. bovis*, the top enriched processes were more variable than in *B. bigemina*, including actin dynamics (profilin, BBOV_II006000) and kinase activity (nucleoside diphosphate kinase family protein BBOV_III005290; adenylate kinase BBOV_IV002930). In *B. divergens* propagated in bovine erythrocytes the most enriched divergent processes were related to fatty acid metabolism/pyrimidine biosynthesis (cytidine diphosphate-diacylglycerol synthase Bdiv_002810c) and iron transport (GTP-binding protein engA Bdiv_017520c). Taken together, each of these suggest there are subtle differences in metabolism between the parasites in the inferred G phase. There were no specifically enriched terms in *B. divergens* cultured in human erythrocytes.

In the inferred SM phase, the most highly enriched GO-term for *B. bigemina* is nuclear outer membrane-endoplasmic reticulum membrane network (BBBOND_0309310, BBBOND_0401740, BBBOND_0307820, BBBOND_0102060, BBBOND_0210875, BBBOND_0210880), suggesting a species-specific increased role for the endomembrane network during this phase. In *B. bovis,* protein-DNA complex is enriched (BBOV_III007560); these genes are both histone proteins, suggesting an increased importance of chromatin structuring during SM phase for *B. bovis.* Finally, the most highly enriched processes for *B. divergens* is oxidoreductase activity (bovine RBCs) (Bdiv_019910, Bdiv_030660, Bdiv_040430c). The observed differences in *B. divergens* are likely driven by the host cell: for bovine RBCs the enriched genes suggest an increased role of the oxidative pentose-phosphate pathway, while in

human RBCs a shift towards expression of proteins needed for post-transcriptional activity. While parasites clearly all undergo DNA replication during the inferred SM phase, these differences suggest that key processes differ during this phase.

In the inferred MC phase there again appears to be an enrichment for activities involved in the endomembrane system in *B. bigemina*. In *B. bovis* there is an enrichment for regulation of hydrolase activity (putative GTP-ase activating protein for Arf BBOV_IV012060; GTPase activator protein BBOV_IV007530) - both enriched genes work to activate GTPases, which are known to be important regulators of mitosis in other systems [4,5]. The orthologs of these genes in *T. gondii* have recently been shown to localize to the nucleus, with the latter of the two appearing to specifically act in the nucleolus, and may play a role in chromatin formation [6]. Interestingly for *B. divergens* there are very few specific markers for MC phase. Indeed for *B. divergens* (bovine) there were no enriched terms, and only a single gene for human- choline/ethanolamine kinase (Bdiv_020970)- suggesting that there is little deviation in the MC phase outside of the conserved process in *B. divergens.*

Finally, for the inferred C phase, there are no significantly enriched species-specific markers for *B. bigemina.* However, for *B. bovis* there is a marked enrichment for proton-transport (proteolipid subunit c BBOV_II002740; vacuolar ATP synthase subunit d BBOV_II001540). In *B. divergens* (bovine) a single gene is enriched – protein disulfide-isomerase (Bdiv_013520c), which in other systems resides in the endoplasmic reticulum and performs an essential function in forming disulfide bonds during protein folding [7]. In human adapted *B. divergens*, oxidoreductase activity appears to be enriched, driven by upregulation of Bdiv_020470c (cytochrome b5-like Heme/Steroid binding domain containing protein). Together, these results suggest there are only subtle differences in parasites during cytokinesis, which is likely a highly conserved process [8].

Fig H

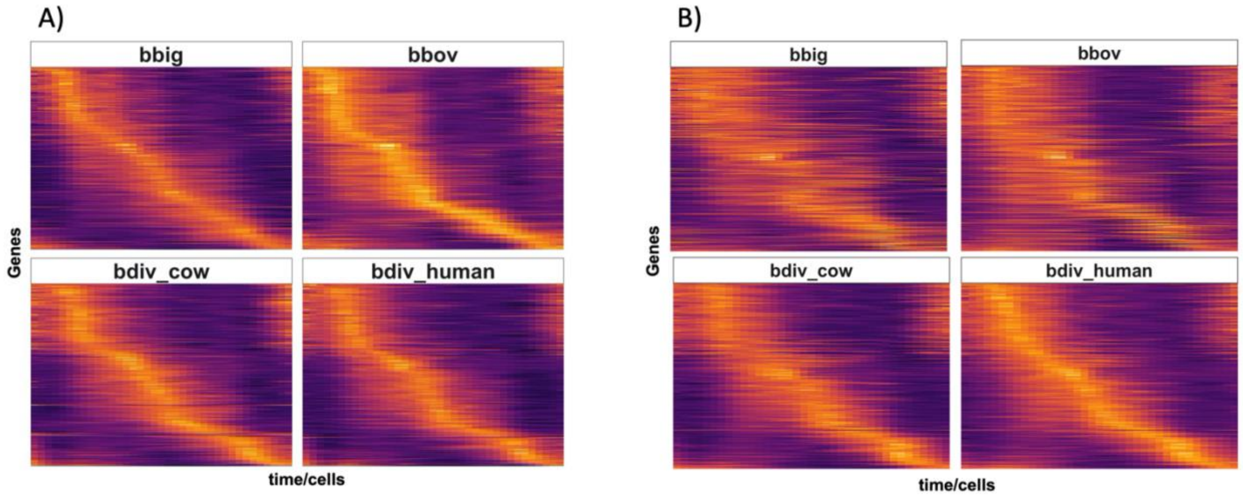

Fig H. Heatmap of gene expression curves of 377 conserved replication cycle markers across all species (main text **Fig 4C**). A) Rows are ordered according to peak expression time in each data sets. B) Rows are ordered according to peak expression time in *B. divergence* in human RBCs. Data and code for generating the figure is available at https://github.com/umbibio/scBabesiaAtlases.

Extended Comparative marker analysis of *B. divergens* in human versus bovine red blood cells

In total, we identified 28 genes across the inferred cell cycle phases that were differentially expressed between B. divergens grown in different host RBCs. Of those, eight were from G inferred state, ten in SM, five in MC, and five in C. In nearly all inferred states, the major upregulation of genes was in parasites grown in bovine RBCs. In G, two upregulated genes were involved in ubiquitin related processes (Bdiv_039000c, Bdiv_017290). Additionally, several genes upregulated in bovine versus human propagated B. divergens in inferred G state were related to transcription and DNA replication (Bdiv_005180c, Bdiv_019130, Bdiv_030460). Progressing into the SM inferred state, again all upregulated genes were observed in parasites cultured in bovine

RBCs. These genes were involved in metabolic processes including lipid metabolism (3-oxo-5-alpha-steroid 4-dehydrogenase family proteins; Bdiv_039610c), pyrimidine biosynthesis (orotidine 5'-phosphate decarboxylase, Bdiv_024970c) and membrane transport (formate/nitrite transporter family protein, Bdiv_007750c). Additionally, there was enrichment for genes related to membrane components (Bdiv_013030c, Bdiv_007750c, Bdiv_017140). The ortholog of the hypothetical protein Bdiv_013030c localized to the Golgi in T. gondii, while the ortholog of Bdiv_007750c appears to be in the plasma membrane [6]. These again seem to underscore differences in nutrient transport and metabolism based on resident host cell. In the MC inferred state, of the five differentially expressed genes, one was specific to human adapted B. divergens - a putative phosphotransferase (Bdiv_020970). The rest of the upregulated genes occurred in bovine adapted parasites, including those involved in cytoskeletal arrangement (Bdiv_038490, Bdiv_016060), and interestingly histone H2Bv (Bdiv_005460c).

Fig I

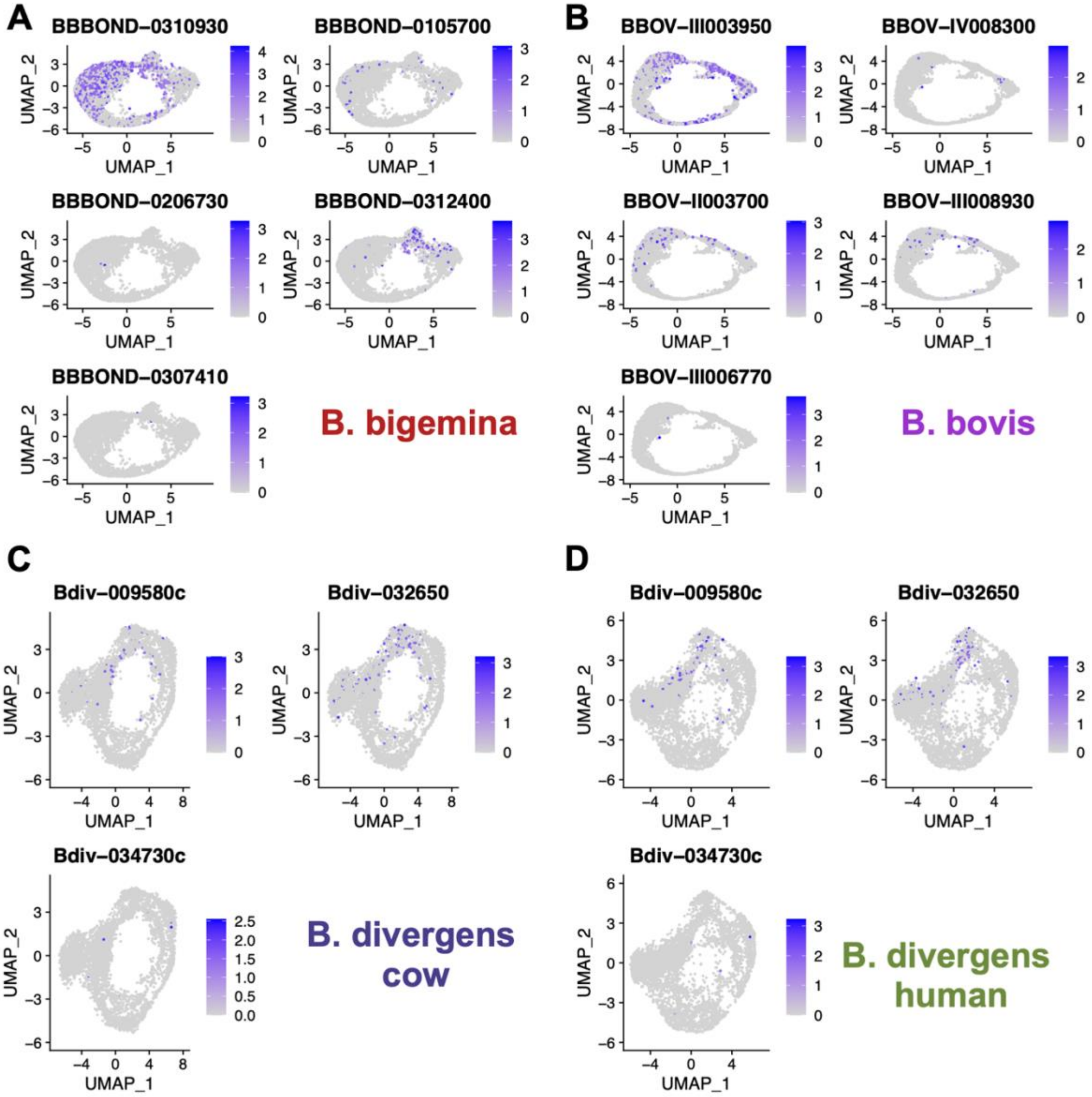

Fig I. Expression of sexual stage markers. Data and code for generating the figure is available at https://github.com/umbibio/scBabesiaAtlases.

Fig J

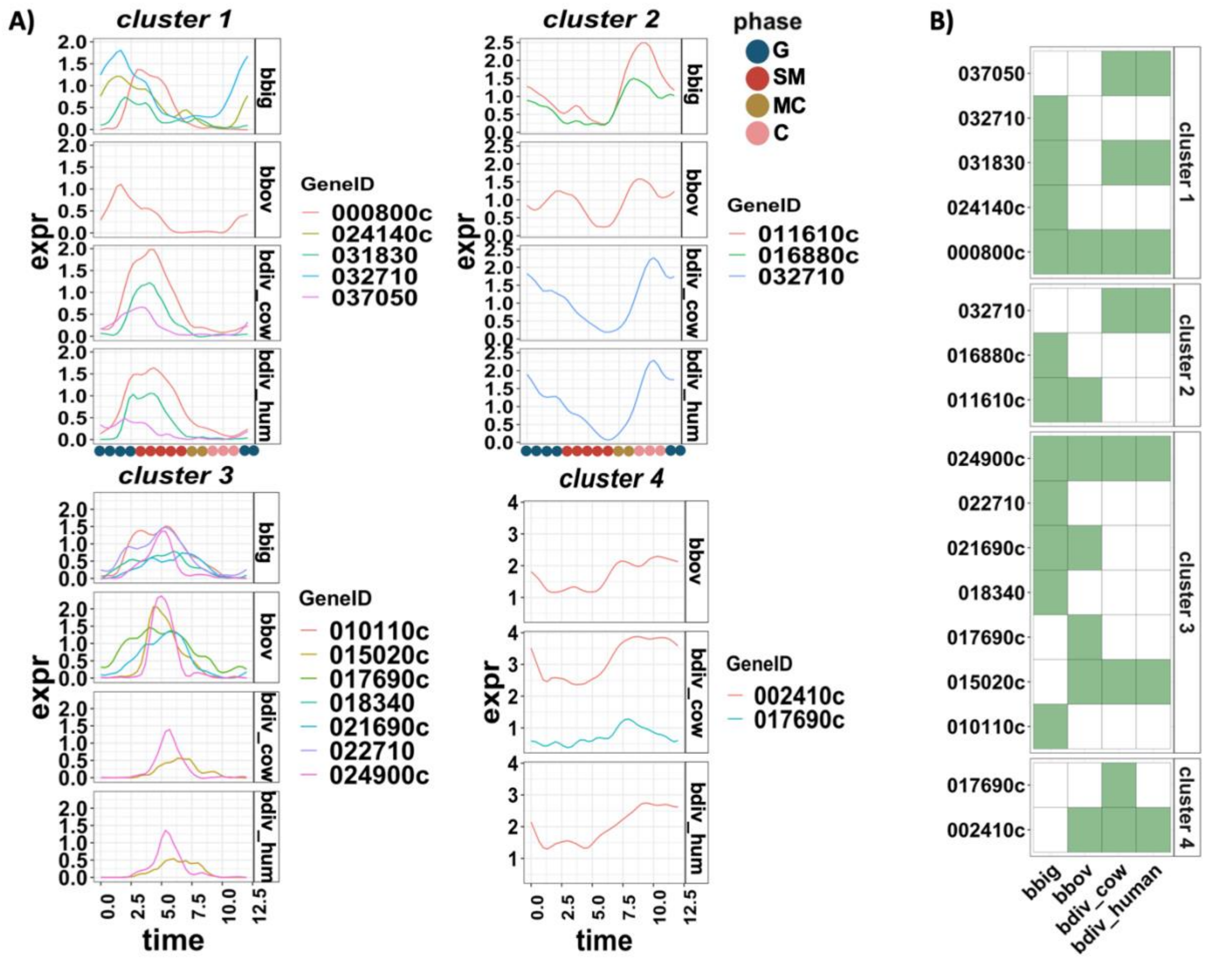

Fig J. Expression profile of Transcription Factors with cyclic pattern (TFs): **(A)** The expression curves of TFs clustered into 4 groups according to their expression similarity, split by species. **(B)** Presence or absence of the gene (rows) in the indicated sample (column). In the heatmap, white indicates that gene is not cyclically expressed in the species, while green indicates that the gene is cyclically expressed in the related species. There are two genes (032710 and 017690c) that switch the clusters. Note: These are clusters of TFs with cyclic expression profile and do not include all TFs. Data and code for generating the figure is available at https://github.com/umbibio/scBabesiaAtlases.

Fig K

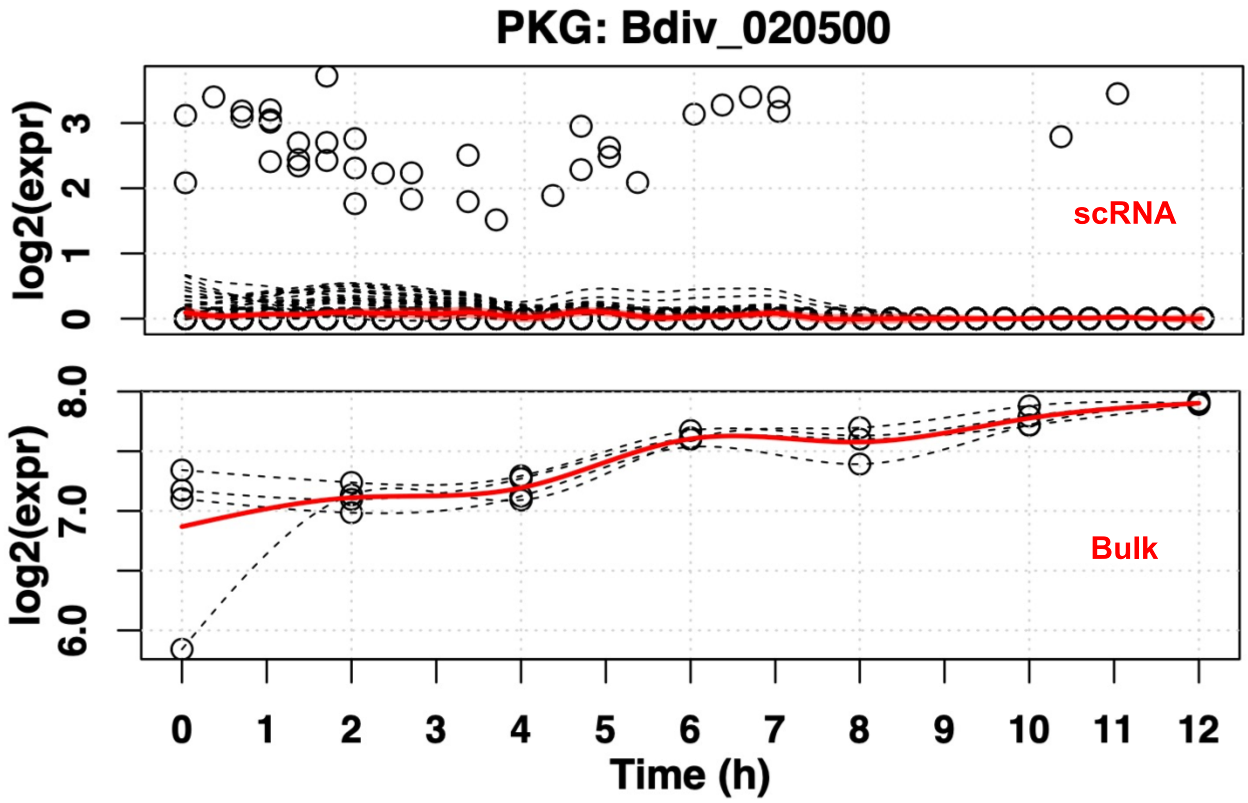

Fig K. Expression of PKG in single cell (**top**) and synchronized bulk (**bottom**). Data and code for generating the figure is available at https://github.com/umbibio/scBabesiaAtlases.

Fig L

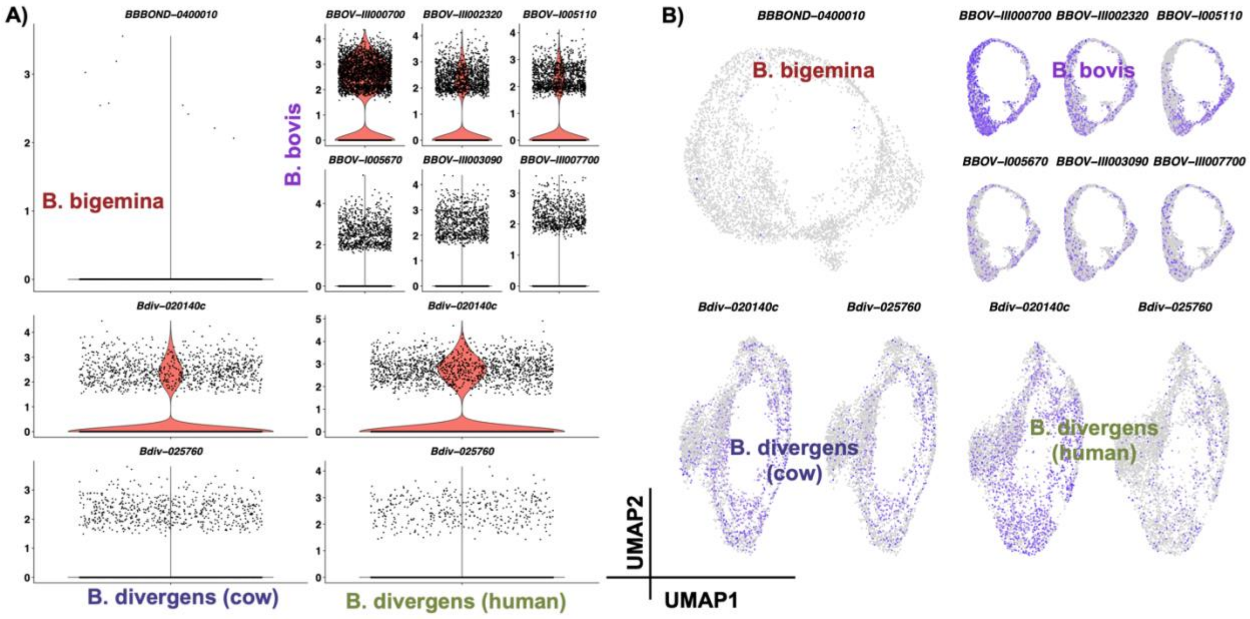

Fig L. Expression of highly expressed (95 percentile) VESA genes: (**left**) violin plots, (**right**) UMAP projection. Data and code to generate the figures are available at https://github.com/umbibio/scBabesiaAtlases. Data and code for generating the figure is available at https://github.com/umbibio/scBabesiaAtlases.

Supplemental References

1. Xue Y, Theisen TC, Rastogi S, Ferrel A, Quake SR, Boothroyd JC. A single-parasite transcriptional atlas of Toxoplasma Gondii reveals novel control of antigen expression. Soldati-Favre D, Kissinger J, editors. eLife. 2020;9: e54129. doi:10.7554/eLife.54129

2. van Dooren GG, Yeoh LM, Striepen B, McFadden GI. The Import of Proteins into the Mitochondrion of Toxoplasma gondii. J Biol Chem. 2016;291: 19335–19350. doi:10.1074/jbc.M116.725069

3. Nozawa A, Ito D, Ibrahim M, Santos HJ, Tsuboi T, Tozawa Y. Characterization of mitochondrial carrier proteins of malaria parasite Plasmodium falciparum based on in vitro translation and reconstitution. Parasitology International. 2020;79: 102160. doi:10.1016/j.parint.2020.102160

4. Chircop M. Rho GTPases as regulators of mitosis and cytokinesis in mammalian cells. Small GTPases. 2014;5: e29770. doi:10.4161/sgtp.29770

5. Miserey-Lenkei S, Colombo MI. Small RAB GTPases Regulate Multiple Steps of Mitosis. Frontiers in Cell and Developmental Biology. 2016;4: 2. doi:10.3389/fcell.2016.00002

6. Barylyuk K, Koreny L, Ke H, Butterworth S, Crook OM, Lassadi I, et al. A Comprehensive Subcellular Atlas of the Toxoplasma Proteome via hyperLOPIT Provides Spatial Context for Protein Functions. Cell Host & Microbe. 2020;28: 752-766.e9. doi:10.1016/j.chom.2020.09.011

7. Laboissière MCA, Sturley SL, Raines RT. The Essential Function of Protein-disulfide Isomerase Is to Unscramble Non-native Disulfide Bonds (*). Journal of Biological Chemistry. 1995;270: 28006–28009. doi:10.1074/jbc.270.47.28006

8. Gubbels M-J, Keroack CD, Dangoudoubiyam S, Worliczek HL, Paul AS, Bauwens C, et al. Fussing About Fission: Defining Variety Among Mainstream and Exotic Apicomplexan Cell Division Modes. Front Cell Infect Microbiol. 2020;0. doi:10.3389/fcimb.2020.00269
